# Integrated Single-Cell Whole-Genome Sequencing and Spatial Transcriptomics Reveal Intratumoral Heterogeneity in Ovarian Cancer

**DOI:** 10.1158/2767-9764.CRC-25-0795

**Published:** 2026-05-04

**Authors:** Rania Bassiouni, Yuxin Jin, Lee D. Gibbs, Jing Qian, Solomon O. Rotimi, Heather Miller, Michelle G. Webb, Seeta Rajpara, Javier Arias-Stella, David W. Craig, Lynda Roman, John D. Carpten

**Affiliations:** 1 Department of Integrative Translational Sciences, Beckman Research Institute, City of Hope, Duarte, California.; 2 Department of Translational Genomics, Keck School of Medicine, University of Southern California, Los Angeles, California.; 3 Department of Pathology, City of Hope Comprehensive Cancer Center, Duarte, California.; 4 Department of Obstetrics and Gynecology, Keck School of Medicine, University of Southern California, Los Angeles, California.

## Abstract

The mortality rate of ovarian cancer remains disproportionately high compared with its incidence. This is partly due to a high level of intratumoral heterogeneity, driven by genomic instability, that promotes disease recurrence and treatment failure. In this study, we describe degrees of heterogeneity revealed by single-cell whole-genome sequencing and spatial transcriptomics (ST) of five late-stage, treatment-naïve primary epithelial ovarian carcinomas, including high-grade serous and clear-cell subtypes. All samples exhibited widespread copy-number (CN) aberrations, with the greatest intraspecimen diversification in regions of CN gain. Diversification was also associated with whole-genome doubling in all samples. In two samples, we identify persistent, clonal pseudodiploid cells evolutionarily consistent with a premalignant phenotype. In multiclonal samples, we interpret clonal evolution in the context of single-cell CN, loss of heterozygosity analysis, and somatic mutations and correlate these with tissue histology and gene expression programs. In one high-grade serous carcinoma, we identify functionally consequential CN alterations that contribute to molecular diversity, cell proliferation, and inflammation in a minor clone that persisted without major expansion alongside a more complex major clone. In another clear-cell carcinoma, we describe a complex evolutionary history, including a spontaneous functional reversion of a *CTNNB1* driver mutation in a secondary clone, which correlated with a switch in oncogenic expression programs. These examples highlight various consequences of genomic instability on clonal heterogeneity and plasticity in ovarian cancer.

**Significance:**

We utilize single-cell DNA sequencing and ST to illustrate the wide extent of intratumoral heterogeneity within late-stage ovarian tumors. We describe several consequences of chromosomal instability, including divergent biology in multiclonal tumors, persistence of a premalignant cell population, and functional reversion of an oncogenic driver mutation.

## Introduction

Ovarian cancer is the deadliest gynecologic malignancy in the United States, with more than half of patients diagnosed with late-stage disease (1). Most ovarian cancers are epithelial in origin and can be classified into four major histologic subtypes: serous, endometrioid, clear cell, and mucinous (2). Among these, high-grade serous ovarian cancer (HGSOC) is the predominant histologic subtype. It is also the most aggressive and has the poorest overall prognosis, with the majority of patients experiencing disease relapse after an initially favorable response to chemotherapy (3). It is estimated that up to 90% of patients with HGSOC diagnosed with metastatic disease will develop total treatment resistance (4).

In contrast to HGSOC, clear-cell ovarian cancer (CCOC) is less common and often presents in younger patients at an early stage, during which it has a more favorable prognosis than HGSOC (5, 6). However, patients with CCOC with late-stage disease have poorer responses to platinum-based chemotherapy and poorer progression-free survival rates than patients with HGSOC (6, 7). Thus, both HGSOC and CCOC are focuses of efforts to improve ovarian cancer outcomes.

In addition to unique histology and clinical characteristics, HGSOC and CCOC are biologically distinct, as evidenced by several studies that have characterized the genomic landscapes of HGSOC and CCOC (8–13). Despite nearly ubiquitous *TP53* mutations, HGSOC exhibits fewer somatic mutations than cancers originating in other tissues (14, 15). Instead, HGSOC is characterized by high-level aneuploidy and recurrent copy-number (CN) alterations (CNA), which are often not directly therapeutically actionable (15). CCOC exhibits a greater diversity in somatic driver variants, dominated by mutations in *ARID1A* and *PIK3CA*, but with *TERT* promoter, *KRAS*, *TP53*, *ATM*, *CTNNB1*, and *PTEN* mutations also occurring at appreciable frequencies (12, 16). Recurrent CNAs are also present in CCOC although with a lower degree of disorder than that seen in HGSOC (13).

Epithelial ovarian cancers (EOC), and particularly HGSOC, are recognized for their intratumoral heterogeneity, which is reflected in both CNA and mutational profiles obtained from multiregion and longitudinal tumor sampling (17–19). Spatial transcriptomics (ST) has been utilized to infer clonal compositions in HGSOC and associated biology in a histologic context (20). However, CN inferred from gene expression data cannot be assessed with confidence at high resolution. Single-cell whole-genome sequencing (scWGS) provides the most direct method to assess CNA heterogeneity and resolve subtle clonal structures that would otherwise be obscured (21–24), but scWGS alone does not describe tumor biology.

In this study, we integrate scWGS with ST analysis of five ovarian cancer samples to assess the biological manifestations of clonal diversity. From scWGS, we assess CN and somatic mutations at the single-cell and cluster level to identify clonal CNAs, whole-genome doubling (WGD) events, and pseudodiploid cells. In multiclonal samples, we infer tumor evolution in the context of CNAs, driver mutations, and loss of heterozygosity (LOH). From matched ST data, we demonstrate that gene expression programs controlling metabolism, cell proliferation, and inflammation correspond to clone-specific CNAs identified in scWGS. We also report, for the first time, a spontaneous reversion of an oncogenic *CTNNB1* driver mutation in a secondary clone and associated compensatory gene expression programs. These cases highlight the myriad contributions of chromosomal instability to intratumoral heterogeneity and the insights that can be gained from the integration of high-resolution genotypic and phenotypic information.

## Materials and Methods

### Samples

Tissue specimens were obtained as optimal cutting temperature–embedded frozen blocks from the University of Southern California Keck School of Medicine Gynecological Tissue and Fluid Repositories under a protocol approved by the Institutional Review Board (#HS-18-00948). The repository also provided matched peripheral blood buffy coat where available. Patients provided written informed consent for sample collection. Samples were all EOC from female patients with varied histologic subtypes: three HGSOC, one CCOC, and one predominantly CCOC mixed with endometrioid. Subtype information was obtained from pathology reports and confirmed by contemporary review. All tumors sampled were high stage (≥IIIC) and high grade. All samples were assessed for tumor adequacy and contained >60% tumor by area in regions sampled. Sample information is summarized in Supplementary File S1.

### DNA extraction and whole-exome sequencing

Genomic DNA was extracted from buffy coat using the DNeasy Blood & Tissue Kit (QIAGEN) according to the manufacturer’s protocol. Tumor DNA was extracted from ten 10-micron scrolls of frozen tumor tissue using the AllPrep Mini Kit (QIAGEN) according to the manufacturer’s protocol. DNA quantity and quality were assessed to ensure sufficiency for library preparation. Following enzymatic fragmentation of the DNA, dual-indexed sequencing libraries were prepared using the SureSelect Low Input Target Enrichment System with SureSelect XT Human All Exon V6 target enrichment probes (both Agilent Technologies). Pooled libraries were sequenced at 2 × 150 on a NovaSeq 6000 instrument (Illumina). Raw sequencing data were demultiplexed using bcltofastq (version 2.20.0.422), and FASTQs were aligned to the human reference genome GRCh38 using bwa mem (version 0.7.17, RRID:SCR_010910) to generate binary alignment map (BAM) files. Whole-exome sequencing (WES) was only performed for sample OV440, for which excess tissue material was available, to benchmark the scWGS described subsequently.

### Bulk CN analysis

The sequenza R package (version 3.0.0, RRID:SCR_016662) was used to process the paired tumor/normal BAMs against a reference genome track (GRCh38) and generate allele-specific CNs (25). A wiggle track file for the reference genome was first created using a window of 50 bp. Sequenza then processed the tumor/normal pair by tabulating reads per genomic bin, followed by segmentation to identify regions of the genome with aberrant CN values. Sequenza output was used to plot CN, depth ratios, and allele frequencies across the genome.

### Nuclear isolation for single-cell DNA sequencing

Single nuclei were isolated from frozen tissue sections as recommended by 10x Genomics (Document CG000167, Rev A). Briefly, tissue sections were thawed on ice, lysed, and physically homogenized with a pestle. Following a dual-step centrifugation, the recovered nuclei were washed in PBS with 0.04% BSA and passed through a 40-micron strainer to remove aggregates. Nuclei were stained with ethidium homodimer-1 and counted with a Countess II FL Cell Counter (Invitrogen).

### scWGS

Single nuclei were processed with Chromium Single Cell DNA Reagent Kits (10x Genomics) according to the manufacturer’s protocol (Document CG000153, Rev B-C). Briefly, individual nuclei are partitioned into single droplets of a hydrogel matrix, within which the nuclei are lysed. This droplet containing denatured genomic DNA is then coencapsulated with a gel bead containing hexamer primers and a unique 16-nucleotide barcode, allowing amplification and unique indexing of DNA from individual cells. The encapsulation is then dissolved, and barcoded DNA is pooled and used to generate Illumina-compatible sequencing libraries. Following quality assessment, libraries were sequenced at 2 × 100 on a NovaSeq 6000 instrument (Illumina).

### Single-cell CN calling and metrics

Sequencing data were processed with the Cell Ranger DNA pipelines (version 1.0.0, 10x Genomics, RRID:SCR_023221). Demultiplexing was performed with *cellranger-dna mkfastq* and was followed by *cellranger-dna cnv* for alignment to the human reference genome GRCh38, cell identification, and CN estimation. The number of cells detected in the samples ranged from 532 to 1,437. An average of ∼1.6B mapped, deduplicated cell-associated reads were processed per sample, for a mean of ∼1.75M reads per cell, sufficient for detecting CN event sizes of ∼1 MB. The median effective reads per MB ranged from 420 to 786. scWGS metrics per sample can be found in Supplementary File S1.

### Postprocessing of single-cell CN calls

To generate a high-quality dataset, we filter both CN calls and cells in a multistep fashion. First, noisy cells and low-quality calls were removed from the dataset (“Filtering of single-cell CN calls”). The remaining cells were subjected to clustering analysis to reveal major CN profiles (“Clustering and subclustering of CN profiles”). Clusters comprising degraded DNA, likely representing apoptotic cells, were identified and removed. Tumor CN profiles were then used to identify and remove possible doublets and computational artifacts (“Removal of doublets and artifacts”). The remaining cells were then subjected to a final round of clustering and subclustering.

### Filtering of single-cell CN calls

Cells were classified as noisy and removed from the data if they met either of two criteria: (i) The ploidy confidence of the cell was low, or (ii) the Depth Independent Median Absolute deviation of Pairwise Differences (DIMAPD) score of the cell—a measure of bin-to-bin variation—was higher than the threshold equivalent to a *P* value of 0.1 when a Gaussian distribution was fit to the data. This DIMAPD filtering is more stringent than the default performed by Cell Ranger, to provide confidence that we were removing cells in the process of active DNA replication, which may confound further analysis. In the remaining cells, individual CN calls per 20 KB bin were assessed for quality, and calls with quality scores <15 were removed. Finally, regions of the genome with mappability <90% were removed. Following this process, the clean data were organized in a matrix of CN calls per 20 KB bin per cell barcode.

### Clustering and subclustering of CN profiles

We employed a clustering strategy based on the one described by Velazquez-Villarreal and colleagues (26). CN calls for 500 sequential bins were aggregated to generate a mean ploidy value at 10 MB resolution in each cell, using the GenomicRanges R package (version 1.50.2, RRID:SCR_000025; ref. 27). The cells were then subjected to maximum likelihood genetic clustering using the R package adegenet (version 2.1.10, RRID:SCR_000825; ref. 28). Data were clustered at all values of *k* up to *k* = 20, and the Bayesian information criterion (BIC) and Akaike information criterion (AIC) were evaluated. Clustering solutions selected based on BIC reflected underclustering, whereas AIC better captured heterogeneous populations (Supplementary Fig. S1). AIC was therefore used to select *k* for clustering. Cells in each cluster were then subjected to a second round of clustering by the same method to determine subclusters. Barcodes retained in each sample and their cluster assignments can be found in Supplementary File S2. Discriminant analysis of principal components (DAPC) was performed using the adegenet package to assess the relationships between determined clusters and subclusters. Euclidean distance of cell pairs within clusters was calculated with the amap R package (version 0.8-19).

### Removal of doublets and artifacts

Following a first round of clustering, tumor clusters that represented a pre-WGD profile were identified and used to generate baseline CN profiles. OV511 and OV594 contained two baseline profiles representing distinct clones. A profile representing a diploid–tumor doublet was then generated by adding two across the baseline tumor profiles. In OV511 and OV594, a tumor–tumor doublet profile was also generated by summing both baseline profiles. Cells matching the doublet profiles were removed from the dataset. We then removed cells that may represent artifacts of CN overfitting by ensuring that cells contained a 10 Mb region with an odd CN, following the strategy of McPherson and colleagues (24). Final assignments and metrics for each barcode per sample can be found in Supplementary File S2.

### Determination of haplotype-specific single-cell CN

From scWGS, the diploid cell cluster was identified in each sample and used as a matched-normal for germline variant calling as follows. The sample BAM file was used to create a mini-BAM containing only diploid cells by adapting the script provided by Velazquez-Villarreal and colleagues (26). The diploid mini-BAM was used to generate a germline variant call format file using GATK HaplotypeCaller (version 4.1.8.0, RRID:SCR_001876; ref. 29). Eagle2 (version 2.4.1, RRID:SCR_015991; ref. 30) was then used to estimate haplotype phase, and autosomal heterozygous single-nucleotide polymorphisms (SNP) were identified using bcftools (version 1.17, RRID:SCR_005227; ref. 31).

The Copy-number Haplotype Inference in Single-cell by Evolutionary Links (CHISEL) algorithm (RRID:SCR_023220; ref. 32) was then used to compute phased single-cell CN. Single-cell read depth ratio (RDR) was calculated across 10 Mb bins, and B-allele frequency (BAF) was calculated across 50 Kb bins for each sample. Cells previously classified as noisy or doublets were removed manually from the RDR and BAF datasets, followed by CHISEL-calling of allele- and haplotype-specific CNs in the remaining cells. LOH was inferred from phased haplotypes. We consider as LOH any region that lacks representation of one allele, which may manifest as hemizygosity, copy-neutral LOH, or LOH with high-level gain of the remaining allele. For assessment of BAF at the cluster level, average BAF at identified SNP positions was binned into 100 Kb blocks to smooth out the noisiness inherent to single-cell data. Allele-specific CN was also used to determine WGD using the guidelines established by Bielski and colleagues (33). Namely, the fraction of the genome with a major allele CN greater than or equal to 2 was determined per cell; a fraction greater than 0.5 indicates WGD.

### Somatic variant calling

Somatic single-nucleotide variants, insertions, and deletions were identified from scWGS at the cluster level using Strelka2 (version 2.9.2, RRID:SCR_005109) in tumor–normal mode (34). Using mini-BAMs (see “Determination of haplotype-specific single-cell CN”), tumor clusters were assessed against matched diploid clusters. Somatic variant calling was performed with default parameters against the GRCh38 reference genome. Variants were annotated using bcftools (version 1.14) against the Single Nucleotide Polymorphism Database build 154 database. Variant effects were annotated and converted to Mutation Annotation Format using vcf2maf (version 1.6.22) with the Variant Effect Predictor database (RRID:SCR_007931; refs. 35, 36). Variants predicted to be pathogenic were visually inspected using Integrative Genomics Viewer (version 2.14.1, RRID:SCR_011793; ref. 37).

### CN visualization

Single-cell CN was visualized on heatmaps, in which each row corresponded to a cellular barcode and each column corresponded to a 10 MB bin using the ComplexHeatmap R package (version 2.14.0, RRID:SCR_017270; ref. 38). The presence or absence of LOH, as determined by CHISEL, was similarly visualized. Heatmaps were organized by and annotated with cluster and subcluster information. Pseudobulk CN line plots were created with the R package ggplot2 (version 3.4.2, RRID:SCR_014601; ref. 39) and reflect the average ploidy value at each 10 MB bin, with shaded regions depicting SD where appropriate. CN line plots paired with chromosome ideograms are displayed at 1 Mb resolution and were created with the R package karyoploteR (version 1.24.0, RRID:SCR_021824; ref. 40).

### Phylogenetic analysis

Phylogenetic analysis was performed based on the strategy employed by Minussi and colleagues (21). Pairwise Manhattan distance was calculated on CN values per cell (in 1 Mb bins) or per subcluster (mean value at 20 Kb bins), using the amap R package. Phylogenetic analysis was then performed with the balanced minimum evolution algorithm in the R package ape (version 5.7-1, RRID:SCR_017343; ref. 41). Trees were rooted by specifying a diploid cell or (sub)cluster as the outgroup and then visualized with the R package ggtree (version 3.6.2, RRID:SCR_018560; ref. 42). For visualization purposes, the diploid clusters were displayed in negative distance space.

### Visium ST

Archival, mounted, and hematoxylin and eosin–stained formalin-fixed, paraffin-embedded tissue was utilized with the Visium CytAssist Spatial Gene Expression assay (10x Genomics). Hardset coverslips were first removed as described in 10x Genomics Demonstrated Protocol CG000518 (Rev C). The Visium assay was then performed following the manufacturer’s protocol, using 11 mm × 11 mm capture slides. Following quality assessment, libraries were sequenced (2 × 100) on an Illumina NextSeq 2000 instrument to generate a minimum of 25,000 read pairs per spot under tissue. Full metrics can be found in Supplementary File S1. Sample OV773 was not subjected to Visium analysis due to sample scarcity.

Raw sequencing data were processed with the *spaceranger mkfastq* and *spaceranger count* pipelines (version 3.0.1, 10x Genomics, RRID:SCR_025848) as described previously (43). The human reference genome GRCh38 was used for alignment. Raw count data were imported into the R package Seurat (version 4.3.0.1, RRID:SCR_016341) and normalized with the SCTransform method from the R package sctransform (version 0.3.5, RRID:SCR_022146, refs. 44, 45).

### CN inference from ST data

The R package infercnv (RRID:SCR_021140; v. 1.18.1; ref. 46) was used to infer CN profiles as follows. Normalized count data were first subjected to ESTIMATE analysis using the R package estimate (version 1.0.13; ref. 47) to determine a tumor purity score for each spot. By comparing tumor purity scores with histologic annotation, we determined that a tumor purity score of 0.7 marked a reasonable threshold to distinguish tumor spots. Therefore, spots with a tumor purity score <0.7 were considered reference spots, whereas spots with tumor purity >0.7 were considered tumor and provided to inferCNV as observations. Raw counts were then analyzed with inferCNV in subcluster mode, with Hidden Markov Model (HMM)-determined CN predictions, to identify CN events in the observation spots relative to the reference spots. Tumor subgroups were determined by inferCNV using the Leiden algorithm.

### Gene set enrichment analysis

Gene set enrichment analysis (GSEA) and single-sample GSEA were performed using GenePattern software (RRID:SCR_003201; ref. 48) as previously described (43). Analysis was performed against the Hallmark gene sets (49) and custom positional gene sets. Cell-cycle phase determination was predicted with Seurat’s gene set–based CellCycleScoring feature.

## Results

### Characteristics of clinical samples

We obtained frozen tumor tissue from five EOC cases. Samples OV440, OV594, and OV773 are HGSOC; sample OV150 is of mixed CCOC and endometrioid histology; and sample OV511 is CCOC. We selected samples with similar stage and grade reflective of clinically challenging late-stage disease. We sampled cases with diverse patient self-reported race: three patients were Hispanic/Latino (OV440, OV511, and OV773), one was Asian (OV150), and one was non-Hispanic White (OV594). All patients were younger than 63, the median age at diagnosis for ovarian cancer (1). Although we did not select based on *BRCA* status, we had prior knowledge of a germline *BRCA2* E49X mutation in sample OV440. All samples were treatment-naïve and obtained from primary biopsies or surgeries.

### Benchmarking of CN calling across modalities

To evaluate subclonal CN heterogeneity, we subjected isolated nuclei from each sample to individual DNA barcoding and shallow WGS (scWGS) as described in “Materials and Methods.” Approximately 1.7 million deduplicated reads were sequenced per cell, for an average of 541 reads per megabase and an average per-cell depth of 0.07× to 0.14× (Supplementary File S1). CN data and metrics produced from the Cell Ranger DNA pipeline were used to filter out low-quality cells and calls. The final proportion of cells passing all filtering steps ranged from 30% to 60% per sample. Clean data were then subjected to maximum likelihood genetic clustering analysis to resolve clusters and subclusters.

**Figure 1.**
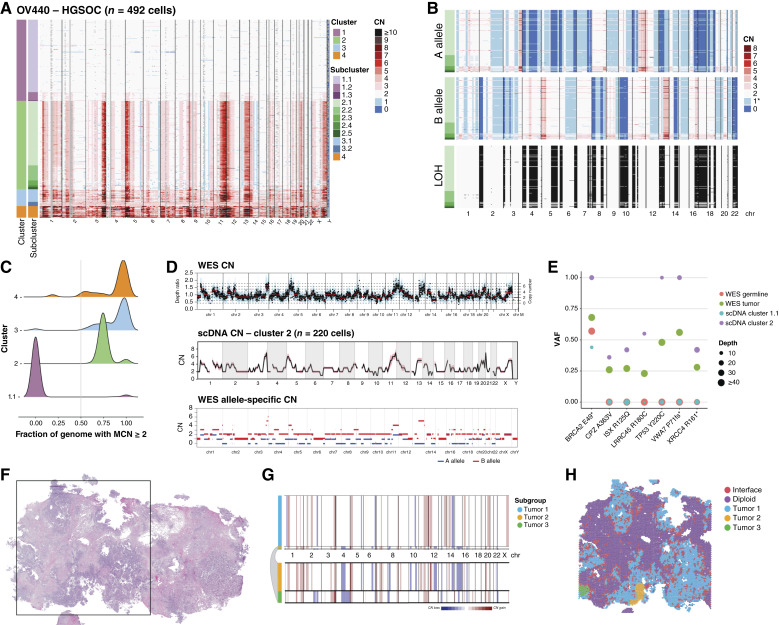
Benchmarking of CN determination across modalities. **A,** CN as determined by scWGS of sample OV440, binned at 10 Mb resolution. Each row displays data from a single cell. Heatmap colors represent absolute CN, whereas gray regions represent unmappable areas or low-quality calls that were removed from the analysis. Chromosomes are delineated and annotated on the *x*-axis. Cells are ordered by cluster and subcluster assignment on the *y*-axis, which were determined by successive rounds of maximum likelihood genetic clustering. **B,** Single-cell allele-specific CNs for OV440 cluster 2, as determined by CHISEL. Heatmap colors match those in **A**; * indicates the expected baseline allele-specific CN of 1 in a heterozygous, diploid cell. LOH is inferred from the A- and B-allele profiles as described in “Materials and Methods” and is represented by black shading. **C,** The fraction of the genome with major CN (MCN) ≥2 was determined per cell and is displayed as a density plot per cluster. Cells with more than half their genome with MCN ≥2 are assumed to have undergone WGD. **D,** (Top) CN as determined by WES of OV440. (Middle) The average CN of cluster 2 from scWGS, binned at 10 Mb resolution. The shaded region around the line represents the SD. (Bottom) The allele-specific CN determined from WES. **E,** The VAFs of select germline and somatic mutations in OV440 as determined from both WES and scWGS. The size of the bubbles represents the number of reads spanning the mutation locus in each sample. **F,** Hematoxylin and eosin–stained tissue of sample OV440. The square represents the 11 mm × 11 mm region sampled by ST. **G,** CNAs inferred from ST using inferCNV. Subclusters were determined by Leiden clustering and are annotated on the left. HMM-based CN prediction was then performed at the subcluster level. Three malignant subgroups were identified; the smaller groups are expanded below the plot for clarity. **H,** Subgroups from (**G**) mapped spatially. Spots labeled “Interface” were determined to be mixtures of malignant and diploid cells (Supplementary Fig. S3). scDNA, single cell DNA.

In sample OV440 (HGSOC), we resolved four clusters (Fig. 1A). Cluster 1 mainly comprises diploid cells, whereas clusters 2, 3, and 4 comprise malignant cells. Subclusters within cluster 2 share the same baseline ploidy but are distinguishable by slight CN differences in chromosomes 1, 2, 8, 13, and X (Fig. 1A). Utilizing the CHISEL algorithm (32), we determined corresponding single-cell haplotype-specific CNs, revealing extensive LOH, including the copy-neutral LOH of the entirety of chromosome 17, encompassing *TP53*, and at chromosome 13q12, encompassing *BRCA2* (Fig. 1B). From CHISEL results, we also determined that cluster 2 had undergone a WGD event, as the main allele is greater than or equal to 2 in over half the genome (Fig. 1C). Clusters 3 and 4 may therefore represent post-WGD diversification and additional WGD, which is a common feature of HGSOC (24).

We benchmarked our scWGS data against CN called from bulk WES. WES-derived total CN and allele-specific CN matched the profile of cluster 2, confirming that clusters 3 and 4 are a minor population in this tumor (Fig. 1D). We also identified somatic variants from scWGS using diploid subcluster 1.1 as a surrogate matched normal-to-tumor cluster 2. Variants included the expected *TP53* mutation, as well as truncating and nonsynonymous mutations in several other genes (Fig. 1E). Variants determined from scWGS were verified in WES, with variant allele fractions (VAF) reflecting that WES sampled a mixture of malignant and nonmalignant cells (Fig. 1E). The germline *BRCA2* truncating mutation and somatic *TP53* Y220C mutation both have a VAF of 1 determined from scWGS, consistent with LOH at both of these loci. With an absolute CN of 2 at both loci, we also infer that the LOH events occurred prior to WGD (Supplementary Fig. S2).

We next assessed the fidelity of CN inferred from ST of OV440 to the ground truth of scWGS (Fig. 1F–H). Application of the inferCNV algorithm revealed three subgroups with distinct CN profiles. The largest of these matched the profile of scWGS cluster 2 although inferCNV could not differentiate between total CNs of 2 and 3 or detect additional WGD. The two other subgroups corresponded to spatially constrained, histologically unique, and transcriptionally distinct groups of cells that likely represent true subclones that were not sampled in scWGS (Supplementary Fig. S3). Thus, CN inferred from ST can generally be matched to scWGS, minding the constraints of sampling bias and algorithm sensitivity.

### Intratumoral CN heterogeneity in EOC

**Figure 2.**
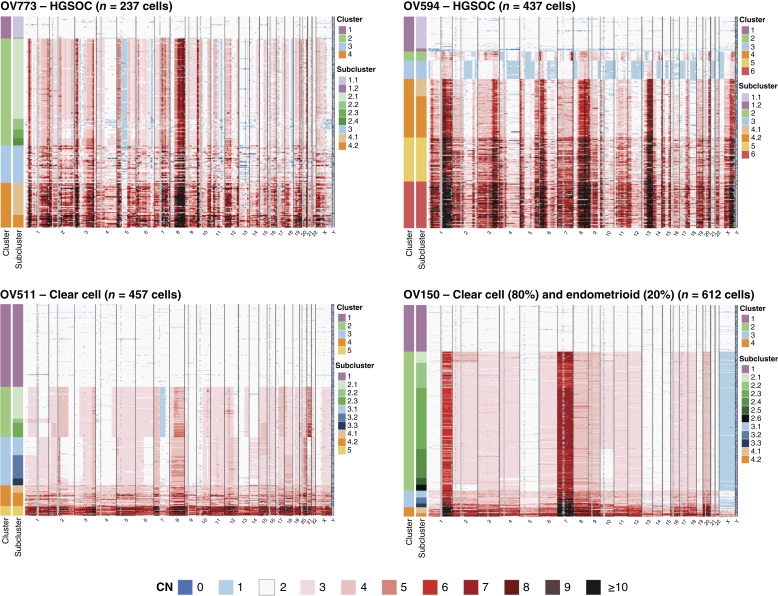
Intratumoral CN variation in additional EOC samples. CN as determined by single-cell DNA sequencing of HGSOC samples OV773 and OV594 and CCOC samples OV511 and OV150. CN is displayed at 10 Mb resolution. Cluster and subcluster assignments are noted on the left. All heatmaps share the color scale displayed below the plots.

We similarly assessed single-cell CN profiles of the four additional EOC samples (Fig. 2). Samples OV773 and OV594 (both HGSOC) exhibited highly disordered genomes and harbored somatic clonal *TP53* mutations with chromosome 17 LOH (Supplementary Fig. S4). OV511 (CCOC) was characterized by arm-level and segmental CNAs, whereas OV150 (primarily CCOC) was characterized by aneuploidy and a lack of focal events, likely reflecting mitotic disjunction. OV150 also harbored a clonal *KRAS* G12V mutation (Supplementary Fig. S5). OV773 and OV150 each contained one clonal profile (cluster 2), whereas OV594 and OV511 contained two distinct major profiles with further subclonal diversity (clusters 2 and 3). We describe the latter two samples in further detail subsequently in this report.

**Figure 3.**
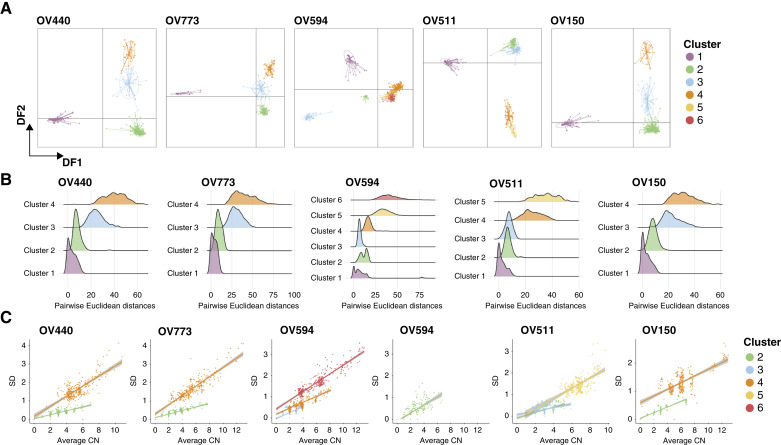
Assessment of cluster diversity. **A,** DAPCs were utilized to describe relationships between clusters in each sample. Plots illustrate discriminant functions (DF) 1 and 2, and points represent individual cells. Loadings corresponding to each DF can be found in Supplementary Fig. S6. **B,** Ridge plot displaying all possible pairwise (cell–cell) Euclidean distances within each cluster and sample. **C,** The relationship between mean CN and SD per 10 Mb genomic bin in tumor clusters. OV594 cluster 2 is shown separately for clarity. Linear regression with a shaded confidence interval is displayed.

We assessed the relationships between identified clusters using DAPCs (Fig. 3A). Baseline ploidy was a primary contributor to intrasample heterogeneity, with WGD driving a main discriminant function in most samples (Supplementary Fig. S6). To examine diversity within clusters, we calculated all unique pairwise distances between individual cells within each cluster assignment (Fig. 3B). Once again, and consistent with recent findings (24), high-ploidy clusters exhibited the greatest intracluster diversity. Interestingly, the greatest intracluster variability occurred in regions of the genome with CN gains, regardless of baseline ploidy (Fig. 3C).

### Assessment of pseudodiploid cells

In all samples profiled, diploid clusters contained individual cells with sparse and low-level CNA. In OV440 (HGSOC), these cells were abundant enough to comprise their own subcluster, 1.2 (Fig. 1A). Such “pseudodiploid” cells have been previously described with varied interpretations. Early scWGS experiments uncovered pseudodiploid cells that did not match the CN profiles of the corresponding malignant cells and therefore seemed unrelated (50, 51). However, a later study described pseudodiploid cells with nonrandom and progressive CN patterns that converged to that of the malignant profile (52). In two of our samples, we find evidence for the latter scenario.

**Figure 4.**
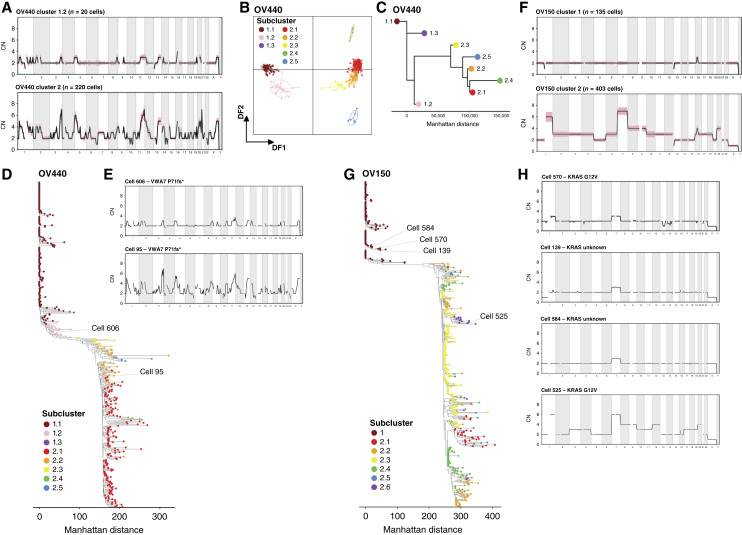
Assessment of pseudodiploid cells in OV440 and OV150. **A,** Average CN at 10 Mb resolution across the genome for subcluster 1.2 and cluster 2 in sample OV440. The shaded region represents SD. **B,** DAPC analysis applied to the subclusters of OV440. **C,** Subcluster-level phylogeny of OV440 based on consensus CN profiles. **D,** Single-cell phylogeny of OV440, based on Manhattan distance and rooted at a true diploid cell. Tips represent cells colored by subcluster assignment. **E,** The CN profile of two single cells annotated in **D** that harbor a *VWA7* frameshift truncating mutation. Cell 606 belongs to pseudodiploid cluster 1.2. Cell 95 is a malignant cell representative of cluster 2. **F,** The average CN profiles of clusters 1 and 2 from OV150. **G,** Single-cell phylogeny of OV150. **H,** CN profiles of single cells annotated in **G**. Cell 570 is a pseudodiploid cell with a *KRAS* G12V mutation, whereas cells 139 and 584 are pseudodiploid cells for which KRAS status could not be ascertained. Cell 525 is a representative malignant cell with a *KRAS* G12V mutation. DF, discriminant function.

In OV440, subcluster 1.2 harbors CN gains at nonrandom sites that match regions that are further amplified in tumor cluster 2 (Fig. 4A). DAPC and phylogenetic analysis confirm that subcluster 1.2 is distinct from the true diploid subcluster 1.1 but is still a great distance from cluster 2 (Fig. 4B–D). Subcluster 1.2 also does not seem to harbor any LOH, including at the *TP53* locus (Supplementary Fig. S7), suggesting that it represents a very early form of malignant transformation. Due to low sequencing depth, we were unable to determine whether these cells harbored the clonal *TP53* Y220C mutation. However, one cell from subcluster 1.2 was found to harbor a truncating frameshift mutation in *VWA7* (Fig. 4D and E), which we had identified as a somatic variant of high VAF in this sample (Fig. 1E). Importantly, *VWA7* lies within a region of chromosome 6p that exhibits clonal copy-neutral LOH in tumor cluster 2. The presence of the mutation in a pseudodiploid cell, therefore, suggests that the mutation preceded the LOH event. This agrees with the paradigm that consequential mutations, including in *TP53*, precede the onset of allelic imbalance in HGSOC (53).

In OV150 (primarily CCOC), no pseudodiploid subcluster was resolved, but several cells within cluster 1 had apparent CNA, particularly in chromosome 7 (Fig. 2). Notably, chromosome 7 also had the greatest magnitude of gain in the malignant cells (Fig. 4F). Phylogenetically, these pseudodiploid cells demonstrated grouped divergence from diploid cells (Fig. 4G). Resolving the *KRAS* G12V variant revealed one pseudodiploid cell in cluster 1 that harbored the mutation (Supplementary Fig. S5; Fig. 4G and H). This cell—cell 570—had a gain of chromosomes 1q and 7 and loss of X. Similar CNA patterns were seen in other pseudodiploid cells for which *KRAS* status could not be ascertained (Fig. 4H). Thus, in both OV440 and OV150, pseudodiploid cells seem related to malignant cells in both somatic variants and CNA site preference.

We also note an interesting observation in the single-cell phylogeny of OV150. Although most subclusters emerge from single or closely grouped branching events, subclusters 2.4 and 2.5 seem to emerge independently at several different positions (Fig. 4G). Subcluster 2.4 is characterized by two copies of chromosome 10, whereas subcluster 2.5 is characterized by two copies of chromosome 4 (Fig. 2). Closer examination reveals considerable variation at chromosomes 1 and 7 within subclusters 2.4 and 2.5 (Supplementary Fig. S8A), suggesting that they derive from multiple other subclusters as a result of aneuploidy in individual cells (Supplementary Fig. S8B). Thus, these may not represent discrete subclones in the evolutionary sense but illustrate the dynamic nature of CNA accumulation in cells prone to aneuploidy.

### Distinct transcriptional profiles in a multiclonal HGSOC

**Figure 5.**
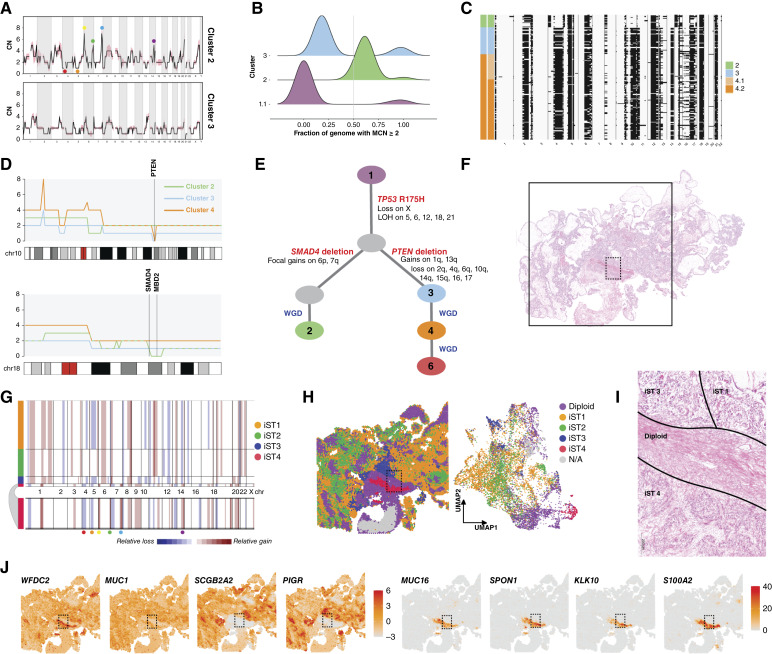
Distinct transcriptional profiles in multiclonal HGSOC. **A,** Average CN of OV594 clusters 2 and 3 derived from scWGS. The shaded area around the line indicates the SD. **B,** Density plot representing the fraction of the genome per cell with a major CN (MCN) greater than or equal to 2. **C,** Single-cell LOH inferred from allele-specific CN for tumor clusters of OV594. LOH is represented by black shading. **D,** CN profiles at 1 Mb resolution across chromosomes 10 and 18 for tumor clusters of OV594. **E,** Hypothesized evolutionary trajectory for OV594. **F,** Hematoxylin and eosin–stained tissue section of sample OV594. The solid square denotes the 11 mm × 11 mm area assessed by ST. **G,** CN inferred from ST data by inferCNV. iST 4 is expanded beneath the plot for clarity. The colored landmarks correspond to those indicated in **A** and help match iST 4 to cluster 2. **H,** Spatial map and UMAP plot of the subclusters determined by inferCNV and shown in **G**. Diploid spots were those used as reference and showed no evidence of CNA. Spots labeled “N/A” did not contain sufficient transcript density and were filtered out by inferCNV. **I,** A magnified region corresponding to the dotted rectangle in (**F** and **H**) labeled with inferCNV assignments. Scale bar corresponds to 200 microns. **J,** Spatially mapped expression for HGSOC-related genes. Scale bars represent Pearson residuals of expression. The dotted rectangle is carried through from the previous panels.

Two distinct malignant CN profiles were resolved in OV594 (HGSOC; Fig. 5A). We consider cluster 2 (*n* = 19 cells) the minor clone, whereas cluster 3 (*n* = 38 cells) is likely the dominant clone and undergoes two rounds of WGD (cluster 4, *n* = 121 and cluster 6, *n* = 96). Interestingly, cluster 2 itself shows evidence for WGD (Fig. 5B) but is not present at any other ploidy. DAPC suggests that clusters 2 and 3 are wholly distinct (Fig. 3A). However, both harbor a *TP53* R175H mutation (Supplementary Fig. S4) and several regions of shared LOH (Fig. 5C). Examination of CN at higher resolution reveals unique additional driver events: focal loss of *PTEN* in cluster 3, which is maintained after WGD, and loss of *SMAD4* in cluster 2 (Fig. 5D). We therefore infer that the clusters arose from the same ancestral clone but with divergent evolutionary trajectories and independent WGD events (Fig. 5E).

To understand the biological context of these distinct cells, we assessed histology and ST data for OV594 (Fig. 5F). The application of inferCNV to ST data predicted four distinct CN profiles, which we refer to as iST (inferred from ST; Fig. 5G). iST 1, 2, and 3 shared similar CN profiles and corresponded to the profile of cluster 3, whereas iST 4 was unique and matched the profile of cluster 2. When plotted spatially and in dimensionally reduced space, iST 4 occupied a discrete location adjacent to, but not intermixed with, the other tumor groups (Fig. 5H). iST 4 also exhibited a solid growth pattern, whereas the region corresponding to iST 1 to 3 had a distinct papillary growth pattern (Fig. 5I). We examined the expression of known ovarian cancer biomarkers in the iST groups (Fig. 5J). All tumor regions expressed *WFDC2* and *MUC1*. However, only iST 1 to 3 expressed *SCGB2A2* and *PIGR*. Interestingly, only iST 4 expressed *MUC16*, also known as CA125, the most widely used clinical diagnostic serum marker for ovarian cancer (54). iST 4 also exclusively expressed *SPON1*, *KLK10*, and *S100A2*—all of which have been proposed as prognostic markers in EOC (55–57).

### Identifying consequential CNA in a HGSOC minor clone

**Figure 6.**
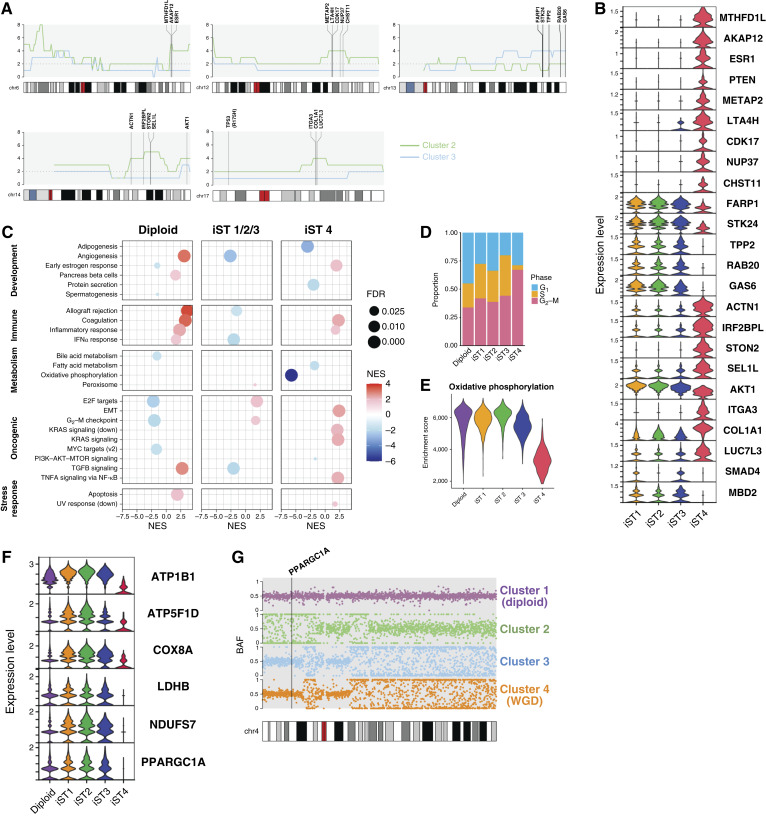
Consequential CNA in a HGSOC minor clone. **A,** CN profiles for OV594 clusters 2 and 3 across chromosomes 6, 12, 13, 14, and 17. Plots are shown at 1 Mb resolution, and select genes are annotated. **B,** Log-transformed expression of genes annotated in **A** in the iST groups defined in Fig. 5F. iST 1, 2, and 3 correspond to cluster 3, and iST 4 corresponds to cluster 2. **C,** Gene set enrichment analysis was performed against the Hallmark gene sets to characterize biological processes in iST groups. Only results with a normalized enrichment score (NES) >1.5 and FDR *q* value < 0.05 are displayed here. **D,** Cell-cycle states of spots assigned to iST groups as a proportion of the total number of spots in each group. **E,** Summary of per-spot enrichment scores in each tumor subgroup for the Hallmark oxidative phosphorylation gene set, as determined by single-sample GSEA. **F,** Log-transformed gene expression of representative nuclear-encoded oxidative phosphorylation genes. *PPARGC1A* encodes the transcriptional coactivator PGC-1α. **G,** BAF in 100 Kb blocks across chromosome 4 for clusters 1–4 of OV594. A BAF of 0.5 reflects allelic balance and heterozygosity, whereas BAFs approaching 0 and 1 reflect LOH. The genomic location of *PPARGC1A* is annotated.

We next examined whether unique CN alterations in the clones of OV594 may be driving functional consequences. In addition to the deep deletions of *PTEN* and *SMAD4*, we identified several regions of CNA in clusters 2 and 3 that were associated with gene expression differences in ST data (Fig. 6A and B). Cluster 3 harbored a gain of a gene-dense portion of chromosome 13, including genes linked to cell-cycle regulation (*TPP2*, *RAB20*; refs. 58, 59), cell survival (*GAS6*, *STK24*; refs. 60, 61), and cell motility (*FARP1*; ref. 62). Among cluster 2–specific gains is an amplification of 6q25.1, containing *ESR1*—an uncommon event in ovarian cancer that is associated with strong protein positivity and potential response to antihormone therapy (63). GSEA also revealed a supportive significant enrichment of early estrogen response genes in iST 4 (Fig. 6C).

Other regions of gain in cluster 2 contained genes linked to cell proliferation and invasion (*METAP2*, *ACTN1*, *ITGA3*, *COL1A1*, *NUP37*; refs. 64–68), drug resistance (*AKAP12*, *COL1A1*; refs. 69, 70), and inflammation (*ITGA3*, *LTA4H*; refs. 71, 72). These genes and related gene sets were significantly enriched in iST 4, including programs for inflammatory response and epithelial–mesenchymal transition (EMT; Fig. 6C). iST 4 also comprised a greater proportion of cells in the G_2_–M-phase (Fig. 6D). Cell-cycle arrest at either G_1_ or G_2_–M has been linked to EMT in cancer and other pathologies (73–75).

GSEA also revealed an absence of oxidative phosphorylation in iST 4 (Fig. 6C and E). Assessment of gene expression of individual electron transport chain components reveals a marked deficiency in iST 4 (Fig. 6F), suggesting a regulatory defect. Expression of nuclear-encoded oxidative phosphorylation genes is coregulated and primarily governed by the transcription factors NRF-1 and NRF-2 and the coactivator PGC-1α (76–78). Both *NRF1* and *NFE2L2*, encoding NRF-1 and NRF-2, respectively, were expressed at low levels throughout the tissue (Supplementary Fig. S9). However, *PPARGC1A*, encoding PGC-1α, was absent in iST 4 (Fig. 6F). Reassessment of scWGS reveals LOH at the *PPARGC1A* locus on chromosome 4p specifically in cluster 2 (Fig. 6G). We can therefore hypothesize that a clone-specific *PPARGC1A* inactivating event, such as hypermethylation or an undetected deleterious mutation, promotes an oxidative phosphorylation deficit in those cells.

### Driver mutation reversion in a multiclonal CCOC

**Figure 7.**
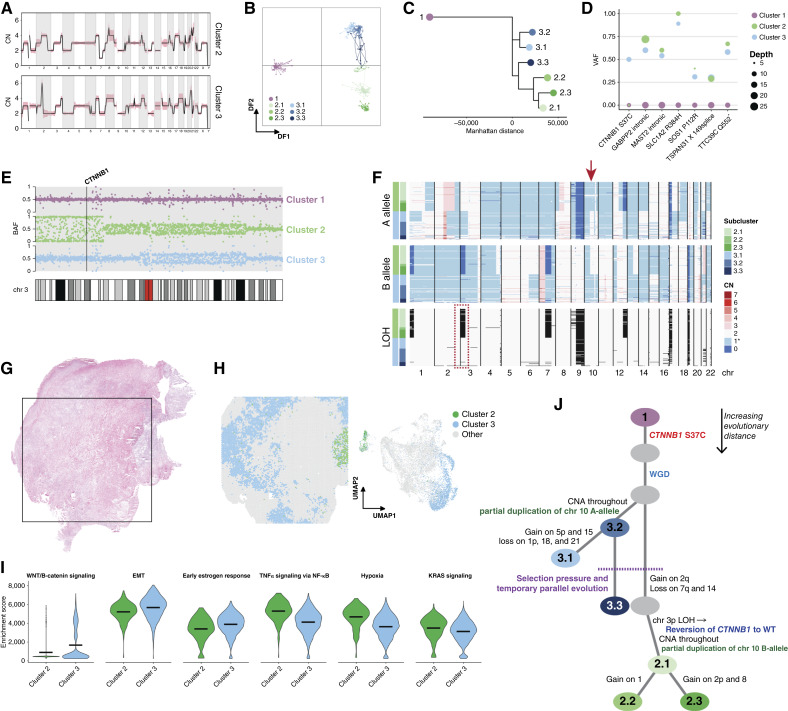
Unique evolutionary history in a multiclonal CCOC. **A,** Average CN profiles for clusters 2 and 3 of OV511. Shading represents SD. **B,** DAPC analysis applied to the subclusters of OV511. **C,** Subcluster phylogeny of OV511. Cluster 1 was specified as the outgroup and is displayed in negative evolutionary space for visualization purposes. **D,** VAFs for select somatic mutations shared by clusters 2 and 3 of OV511. **E,** BAF in 100 Kb blocks across chromosome 3 for OV511 clusters 2 and 3. The *CTNNB1* locus is annotated. **F,** Single-cell allele-specific CNs and inferred LOH for sample OV511. LOH is represented by black shading. The arrow marks the location of a mirrored convergent evolution event on chromosome 10. At this region, cluster 2 has two copies of the B allele and one copy of the A allele, whereas cluster 3 has one copy of the B allele and two copies of the A allele. Both clusters thus have an absolute CN of 3. The dotted box highlights the LOH on chromosome 3 that results in *CTNNB1* wild-type (WT) reversion. **G,** Hematoxylin and eosin–stained tissue section of OV511. The square denotes the 11 mm × 11 mm area captured by the Visium assay. **H,** Spatial map and UMAP of ST spots determined to represent clusters 2 and 3 by positional gene set enrichment. **I,** Aggregate enrichment scores for ST annotations in **H**, as determined by single-sample GSEA against the Hallmark gene sets. The mean of each group is annotated by a horizontal line. All plots shown have a Bonferroni-corrected *P* value < 0.0001. **J,** Hypothesized evolutionary trajectory for sample OV511. Somatic *CTNNB1* mutation is the oncogenic driver and is followed by a WGD event. Two main branches are distinguished by unique CNA, including mirrored allelic imbalance on chromosome 10. A parallel evolution event drives similar subclonal alterations before cluster 2 diverges with a unique set of CNAs, including loss of the *CTNNB1* mutation. DF, discriminant function.

Tumor OV511 (CCOC) contained two distinct major CN profiles, clusters 2 and 3, with several shared characteristics, including WGD and further CNA of chromosomes 5, 7p, and Xq (Fig. 7A; Supplementary Fig. S10). However, cluster 2 exhibited unique gains of regions of 2q, 4q, and 13q and loss within 7q. Cluster 3 had a gain of 1q, a unique focal amplification within 2q, and gain of chromosome 14. Further heterogeneity is seen within subclusters, with most subclusters having at least two distinguishing characteristics. Subcluster 3.3 is notable for sharing several characteristics with cluster 2, including gain in similar regions of 2q, loss at 7q, and two copies of chromosome 14 (Fig. 2). DAPC confirms the intermediacy of subcluster 3.3, as does phylogenetic inference (Fig. 7B and C).

Variant analysis revealed several shared somatic variants with comparable VAF in both clusters (Fig. 7D; Supplementary Fig. S11), reflecting a shared ancestry. A heterozygous *CTNNB1* S37C hotspot mutation was also identified in cluster 3 (Fig. 7D), but no other driver candidates were identified in either clone. As cluster 3 evolutionarily precedes cluster 2 and *CTNNB1* mutations are nearly always clonal events (79, 80), we infer that this is a truncal driver mutation in this tumor. We provide further assessment of the likelihood of a clonal versus subclonal *CTNNB1* mutation in Supplementary File S3. Interestingly, cluster 2 did not harbor the mutation. Assessment of BAF across chromosome 3 indicates LOH at *CTNNB1* in cluster 2, whereas cluster 3 reflects heterozygosity (Fig. 7E). Genome-wide phased CN confirms cluster-specific loss of the B allele at this location (Fig. 7F), as does manual examination of heterozygous germline SNPs within *CTNNB1* introns (Supplementary Fig. S12). This therefore seems to be a spontaneous loss of a driver mutation in a secondary clone via LOH that retained the wild-type allele.

In ST data of OV511, low transcript counts prevented inferCNV from resolving CN profiles. We therefore identified epithelial-majority spots corresponding to clusters 2 and 3 by positional gene set enrichment (Supplementary Fig. S13). As with other samples, the distinct clusters occupied discrete spatial and low-dimensional locations (Fig. 7G and H) and had distinct histologies (Supplementary Fig. S14). Examination of oncogenic pathway expression revealed enrichment of WNT/β-catenin signaling and EMT in cluster 3, consistent with an activating *CTNNB1* mutation (Fig. 7I). This cluster was also enriched in estrogen signaling, which is also linked to β-catenin activity (81). Cluster 2 had greater enrichment of TNFα and KRAS signaling, as well as hypoxia markers, indicating that these expression programs may compensate for *CTNNB1* reversion in this sample.

We can therefore construct a representation of the unique evolutionary history of OV511 (Fig. 7J). Although cluster 3 unambiguously precedes cluster 2, the placement of subcluster 3.3 is confounded by its similarities to cluster 2. From allele-specific CN, we determine that subcluster 3.3 matches cluster 3 in LOH patterns and in the presence of a mirrored convergent evolution event on chromosome 10 (Fig. 7F). Thus, although phylogeny based on absolute CN alone suggests that subcluster 3.3 and cluster 2 share a most recent common ancestor (Fig. 7C), that is biologically unlikely. Instead, we posit that subcluster 3.3 is evolutionarily grouped with cluster 3, but that the emergence of an environmental pressure selected for similar CNA in subcluster 3.3 and the nascent cluster 2. This temporary parallel evolution diverged when cluster 2 acquired further somatic alterations, including the functional reversion of *CTNNB1* to wild type.

## Discussion

Ovarian cancer is notable for its high degree of genomic disorder and intratumoral heterogeneity. Through the integration of ST and scWGS, we describe cell-level and subclonal features that would be indistinguishable from each modality alone.

We describe several shared characteristics of all five samples. All samples contained cells at multiple ploidies, supporting the paradigm established by McPherson and colleagues (24) that mixed WGD multiplicities may exist within one HGSOC tumor and extending that finding to CCOC. We also consistently find that regions of CN gain have the greatest cell-to-cell variability in absolute CN, suggesting that gains accumulate at preferential sites throughout a tumor’s development. This notion is further supported by our finding that CN gains that appear in pseudodiploid cells are preferentially amplified, even after WGD.

Two samples—OV440 and OV150—contained distinctly clonal pseudodiploid cells harboring somatic mutations that matched those in malignant cells, including a *KRAS* G12V driver mutation. Pseudodiploid cells have been described in other cancer types, including colon, breast, gastric, and prostate cancers (52, 82). However, this is the first examination of pseudodiploid single cells in ovarian cancer and the first evidence of pseudodiploid cells harboring a *KRAS* mutation. In the context of ovarian cancer, pseudodiploid cells may reflect the residual presence of serous tubal intraepithelial carcinomas (STIC), early lesions in the fallopian tube, which give rise to HGSOC (83). However, we also report pseudodiploid cells in a sample of clear cell histology, which is not associated with STIC precursors. Generally, the persistence of pseudodiploid cells in late-stage tumors may represent a reservoir of premalignant cells that contribute to treatment escape and disease recurrence.

Two multiclonal tumors provided us the opportunity to infer subclonal evolution and associated somatic events. Sample OV594 was a HGSOC with a major clone and a minor clone of significantly fewer cells. After the acquisition of an initial, shared *TP53* mutation, the two populations diverged significantly. Each further lost an additional tumor suppressor gene: *SMAD4* in cluster 2 and *PTEN* in cluster 3, followed by independent WGD events. In each clone, gene dosage variations were reflected in gene expression differences. In the minor clone, enrichment of transcriptional programs related to EMT, inflammation, and metabolic capacity correlated with CNAs. Based on both scWGS and ST, we conclude that the minor clone persisted in the tumor alongside the major clone despite not undergoing major expansion or further subclonal development. The restricted expression of *MUC16*, or CA125, to the minor clone also has implications for the effect of tumor heterogeneity on clinical diagnostic testing.

The second multiclonal sample, OV511, was a CCOC and comprised diploid cells and two distinct malignant populations. We establish that the two malignant clusters share a common ancestor based on the following evidence: They share identical CN profiles on several chromosomes, they share identical copy-neutral LOH at several loci—indicative of a truncal WGD event—and they share several hundred somatic passenger mutations. On the premise of a shared ancestral clone, we deduce the order of clone emergence from the mathematical evidence of phylogeny analysis and from the logical evidence of LOH patterns, which can be gained but not lost.

The early clone harbored a heterozygous *CTNNB1* S37C mutation, a strong activator of β-catenin signaling and a pan-cancer oncogenic driver (84–86). Surprisingly, the secondary clone did not harbor the mutation but harbored LOH on chromosome 3 encompassing the *CTNNB1* locus. Although we do not have direct evidence of the reversion of this mutation, we can infer that it was a truncal driver for several reasons. Functional driver mutations are nearly always clonal, as they promote clonal sweeps during tumorigenesis (87). Specifically, *CTNNB1* driver mutations are clonal in 96% of ovarian adenocarcinomas that harbor them, and the median number of driver mutations per tumor in this cancer type is one (80). As we also do not find an alternate clonal driver candidate in OV511, we find a clonal *CTNNB1* mutation with subclonal reversion to be strongly supported in this sample.

Although mutation reversions typically involve the restoration of function to tumor suppressor genes, there is precedent for oncogenic mutation reversion. Loss of an oncogenic driver mutation was recently reported in a liver metastasis of an *EGFR*-driven lung adenocarcinoma following treatment with a neopeptide vaccine targeting the mutation (88). In that case, the loss of the driver *EGFR* mutation was also suspected to be caused by LOH resulting from chromosomal instability. The reversion of *CTNNB1* in OV511 is unique in that it occurs at the primary site in a treatment-naïve tumor and is therefore not the result of external selective pressures. Although the accepted paradigm of tumor evolution holds that functional driver gene mutations are highly conserved throughout tumor progression (87), we note that subclonal reversions would not be discernible in bulk or even multiregion sequencing. Such reversions may therefore occur more frequently than expected, simply as a result of chromosomal instability. Although it is likely that reverted cells are quickly outcompeted by the fitter clone, reversions may be viable on a cellular background that allows cells to tolerate the loss of the initial driver mutation. In OV511, this may have been facilitated by potently oncogenic expression programs like TNFα and KRAS signaling.

As the relevance of intratumoral heterogeneity to the clinical course of disease has been well established (89), we can infer clinical consequences of clonal diversity in samples OV594 and OV511. First-line chemotherapy for late-stage EOC is invariably platinum and taxane, which have reduced efficacy in tumors displaying an EMT phenotype (90). We describe a strong enrichment of EMT processes in OV549 cluster 2 and OV511 cluster 3, consistent with *SMAD4* deletion and β-catenin activation, respectively. These cells may therefore contribute to treatment resistance and require alternative targeted therapies. For example, OV594 cluster 2 may benefit from TGFβ inhibition, which has been shown to be effective when *SMAD4* is lost (91), or from antiestrogen therapies targeting the cluster-specific *ESR1* amplification. Similarly, OV511 cluster 3 may be sensitive to β-catenin antagonists or TTK inhibition (92, 93).

It is important that we interpret these results within the context of study limitations. Although CN and mutational profiles provide a great deal of insight into tumorigenesis, epigenetic modifications are also important and often contribute to genomic instability in ovarian cancer (94). Due to a lack of sample material, we were unable to investigate epigenetic silencing events in our samples. We also limit our analysis of mutations to sample- and clone-level events as the aggregated depth of coverage lends greater confidence to the results. We therefore do not identify subclonal mutations although these would be informative of tumor evolution when integrated with CN-defined subclusters.

Our study supports the intrinsic role of genomic variation and heterogeneity in the tumor progression model of ovarian cancer. Although HGSOC is the EOC subtype most typically associated with chromosomal aberrations, we find that CCOC may also harbor complex and consequential genomic events. Examination of single-cell genomes is therefore particularly valuable in cancers driven by chromosomal instability, like EOC, in which biologically significant CNAs may occur in minor cell populations.

## Supplementary Material

Supplementary File 1 Sample-level metadata and assay metrics.

Supplementary File 2 Cell-level single cell whole genome sequencing metrics and cluster assignments.

Supplementary File 3 Assessment of two models of OV511 evolution

Supplementary Figure 1 Clustering of scWGS copy number data

Supplementary Figure 2 Copy number and LOH at chromosomes 13 and 17 in OV440

Supplementary Figure 3 Copy number inferred from OV440 ST data

Supplementary Figure 4 TP53 mutations in HGSOC samples

Supplementary Figure 5 OV150 somatic KRAS mutation

Supplementary Figure 6 Loadings corresponding to DAPC analysis in Figure 3

Supplementary Figure 7 Chromosome 17 LOH in OV440

Supplementary Figure 8 Evolution of OV150 subclusters 2.4 and 2.5

Supplementary Figure 9 Spatial NRF1 and NFE2L2 expression in OV594

Supplementary Figure 10 Shared copy number events in OV511 clones

Supplementary Figure 11 OV511 somatic variants

Supplementary Figure 12 LOH at CTNNB1 SNPs

Supplementary Figure 13 Positional gene set enrichment in OV511

Supplementary Figure 14 Histology of OV511 clones

## Data Availability

Processed single-cell CN and ST data are archived at Zenodo and can be freely accessed at https://doi.org/10.5281/zenodo.17418856. Code for the analysis of single-cell CN data can also be accessed at this Zenodo record. Raw Visium ST data (in the form of BAM files) are freely available through the European Nucleotide Archive at accession PRJEB102074 and can be accessed at https://www.ebi.ac.uk/ena/browser/view/PRJEB102074. Raw scWGS data (in the form of BAM files) are archived with controlled access at the European Genome-phenome Archive at accession EGAD50000002102 and can be accessed at https://ega-archive.org/datasets/EGAD50000002102. Access to the dataset is controlled by the Data Access Committee EGAC50000000834 and governed by policy EGAP50000000783.
