# Supplementary material for: Integrated Single-Cell Whole-Genome Sequencing and Spatial Transcriptomics Reveal Intratumoral Heterogeneity in Ovarian Cancer
Source: Cancer Res Commun. 2026 May 4;6(5):1020–35. doi: 10.1158/2767-9764.CRC-25-0795 (PMC13137417; doi:10.1158/2767-9764.CRC-25-0795)
Supplement: Supplementary File 3 — Assessment of two models of OV511 evolution [file crc-25-0795_supplementary_file_3_supps3.pdf]

Supplemental File 3 – Assessment of two models of OV511 evolution

In the main text, we describe OV511 as likely harboring a clonal *CTNNB1* S37C mutation that is lost in the secondary clone through a loss of heterozygosity (LOH) event. However, we were unable to phase the mutation to confirm that it was on the allele that was lost, which is a limitation of short read sequencing. Thus, we also explore the possibility that the *CTNNB1* mutation in OV511 is a subclonal, rather than clonal, driver mutation. We assess the logic and likelihood of two possible models of OV511 evolution below.

<u>Model 1: Clonal *CTNNB1* mutation</u>

As discussed in the main text, OV511 may be explained by a truncal *CTNNB1* mutation that is lost in cluster 2 via LOH. A simplified representation of this trajectory is shown below (see main Figure 7J for a more detailed representation).

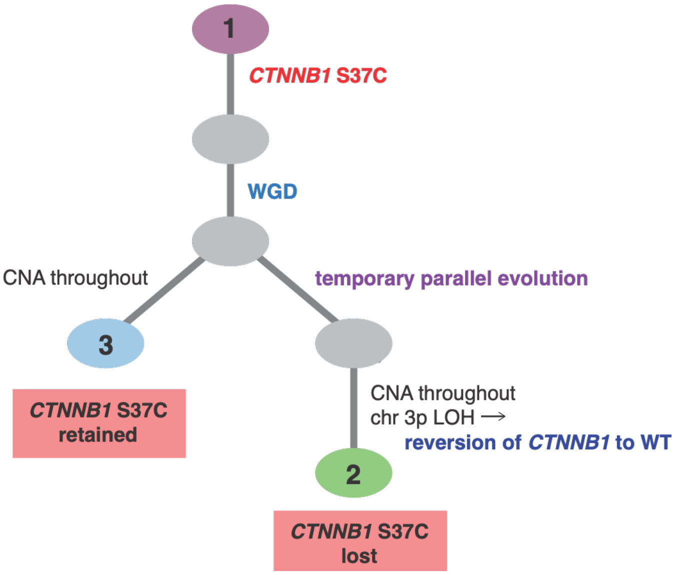

We consider the following points that are demonstrable from our data and described in the main text:

- Cluster 2 and cluster 3 arise from the same ancestral clone (evidenced by copy number, LOH, and SNV analysis)
- Clusters 2 and 3 have undergone a whole genome doubling (WGD) event (evidenced by allele-specific copy number)
- Cluster 3 precedes cluster 2 evolutionarily (evidenced by LOH patterns and phylogeny analysis)

- Cluster 3 harbors a *CTNNB1* mutation that is a known hotspot driver mutation (reviewed in [1, 2])
- Cluster 2 harbors LOH at *CTNNB1*. Specifically, the B allele is lost.

Thus, only one assumption must be made when considering this model:

- The *CTNNB1* S37C mutation sits on the B allele in cluster 3. With a heterozygous genotype and total copy number of 2 at that segment of chr 3p, the probability of this being true is 0.5.

<u>Model 2: Subclonal *CTNNB1* mutation</u>

Another possibility may also explain OV511: the observed *CTNNB1* mutation may be subclonal, only arising in cluster 3. In this model, the clonal driver is unidentified. An illustration of the trajectory is shown below.

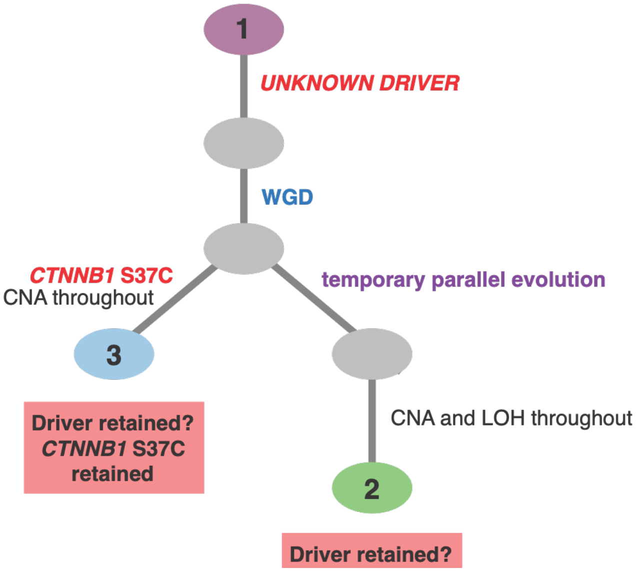

With *CTNNB1* moved to a subclonal branch, a different assumption must be made:

- The truncal driver, which should be present in all cluster 2 and 3 cells, is undetectable in our data.

As described in Materials & Methods, we utilized GATK Haplotype Caller and Strelka2 for germline and somatic variant detection, respectively. Assessment of called variants revealed no alternate driver candidate with a variant allele fraction supporting clonality. With the understanding that shallow sequencing does not provide the depth typically preferred for variant detection, we applied Picard tools [3] (CollectWgsMetrics and

CollectHsMetrics, v. 2.21.1) to cluster mini-BAMs to relate depth and coverage at the cluster level. The metrics are summarized in the table below.

	Mean depth (whole genome)	Mean depth (protein coding regions)	Proportion of genome with 5x depth	Proportion of genome with 10x depth	Proportion of genome with 15x depth
Cluster 1	9.492259	12.960693	0.907317	0.489746	0.09543
Clusters 2 + 3	14.745231	19.811351	0.945723	0.803744	0.493432
Clusters 1 + 2 +3	24.214523	32.772044	0.96083	0.948419	0.896103

We assess the likelihood of a missed germline driver as follows:

- Cluster 1 (179 cells total) has an average read depth of ~13 in protein coding regions. The likelihood of missing a heterozygous germline mutation is therefore very small (~0.5^{13}). Considering that a germline mutation would also be retained in the tumor, the aggregate mean depth of ~33 in cluster 1 + 2 + 3 lends further confidence.
- We also manually examined via IGV the genes most linked to ovarian cancer predisposition (*BRCA1, BRCA2, NTHL1, BRIP1, RAD51C, RAD51D, PALB2, ATM, MLH1, MLH3, MSH2, MSH3, MSH6, PMS1, PMS2, EPCAM, STK11, TP53, CHEK2* [4]) and found no SNVs or indels with clinical evidence for pathogenicity.

We therefore rule out the possibility of an undetected germline diver in OV511. We assess the likelihood of a missed somatic driver similarly:

- In aggregate, clusters 2 and 3 (total of 213 cells) have an average read depth of ~20 in protein coding regions. Because we are assessing a pure tumor population, we would expect to see ample representation of a clonal mutation, especially one that occurred prior to a WGD event.
- To estimate the expected allele fraction of a clonal mutation, we use the average ploidy of cells in clusters 2 and 3, which is 2.8 (Supplemental File 2). In the most conservative scenario, the mutation may only be present in one of 3 copies per cell for an allele fraction of 0.33 distributed across both clusters. We do not find any other driver mutation approaching this allele fraction.
- We also note that approximately 10% of the genome in cluster 2 comprises LOH; with representation of only one allele, the likelihood of missing a conserved driver in these regions is 0.
- In addition to the predisposition genes listed above, we also manually examined several driver genes commonly mutated in CCOC (*ARID1A, PIK3CA, TERT, KRAS, PPP2R1A* [4]) and found somatic driver mutations in none.

We also confirm that there is no somatic deep deletion in either clone. Commonly amplified copy number drivers *MYC*, *PIK3CA*, *ERBB2*, and *TERT* display modest gains of 1-2 copies, attributable to WGD.

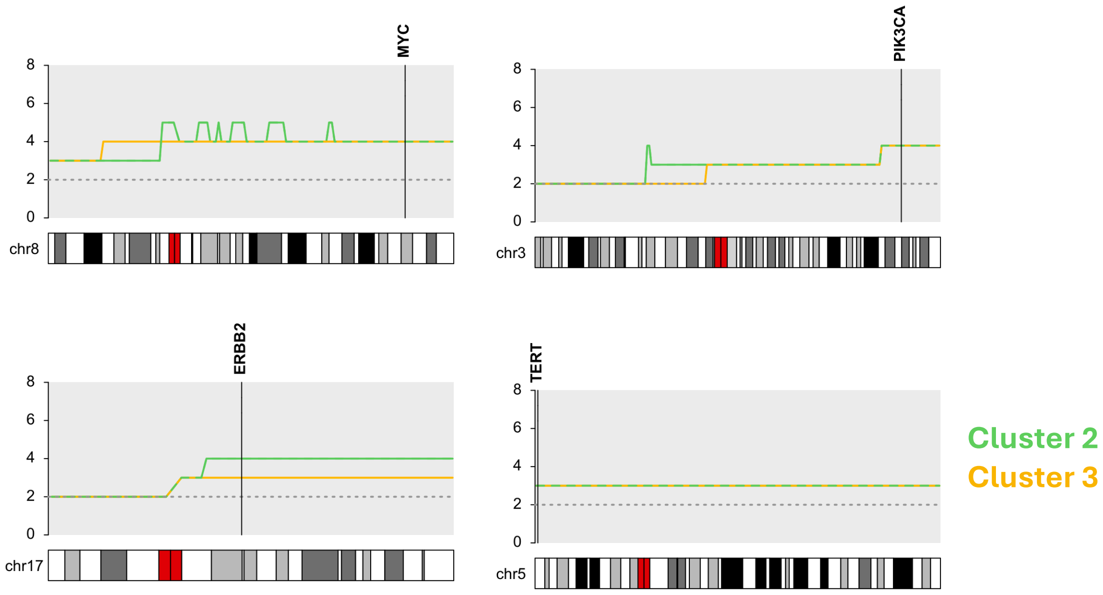

We additionally applied manta (v. 1.6.0 [5]) and AnnotSV [6] to cluster mini-BAMs for structural variant detection and found no driver candidates.

<u>In conclusion</u>

The paradigm of tumor evolution holds that three driver alterations are required to transform a healthy cell into a malignant cell, with each subsequent mutation providing a stronger growth advantage, until a clonal sweep occurs [7]. Functional driver mutations are nearly always clonal, as they provide a strong enough growth advantage to promote a clonal sweep during early tumor development [7]. As *CTNNB1* hotspot mutations are strong activators of oncogenic signaling [8], we would expect them to follow this pattern. Indeed, *CTNNB1* driver mutations are clonal in 88% of tumors that harbor them, and that number rises to 96% in ovarian adenocarcinoma [9]. This notion is also supported by several studies that describe *CTNNB1* hotspot mutations in early-stage cancers [10-12]. In cases where *CTNNB1* mutations have been described as subclonal, such as a HGSOC case profiled by Bashashati et al [13], a clear clonal driver mutation is also detectable (*TP53* in that example). Finally, we consider recent evidence that genome instability may cause driver mutation loss through LOH [14]. Taken together, in the absence of an alternative clonal driver, we find model 1 to fit best with what we know to be true of sample OV511, of *CTNNB1* mutations, and of tumor evolution.

Supplemental References

1. Polakis P. Wnt signaling and cancer. Genes Dev. 2000;14(15):1837-51.
2. Gao C, Wang Y, Broaddus R, Sun L, Xue F, Zhang W. Exon 3 mutations of CTNNB1 drive tumorigenesis: a review. Oncotarget. 2018;9(4):5492-508.
3. Picard Toolkit [Internet]. Broad Institute. 2019. Available from: https://github.com/broadinstitute/picard.
4. Chao A, Huang CY, Yu W, Lin CY, Lin H, Chao AS, et al. Molecular profiling reveals novel therapeutic targets and clonal evolution in ovarian clear cell carcinoma. BMC Cancer. 2024;24(1):1403.
5. Chen X, Schulz-Trieglaff O, Shaw R, Barnes B, Schlesinger F, Kallberg M, et al. Manta: rapid detection of structural variants and indels for germline and cancer sequencing applications. Bioinformatics. 2016;32(8):1220-2.
6. Geoffroy V, Herenger Y, Kress A, Stoetzel C, Piton A, Dollfus H, et al. AnnotSV: an integrated tool for structural variations annotation. Bioinformatics. 2018;34(20):3572-4.
7. Reiter JG, Baretti M, Gerold JM, Makohon-Moore AP, Daud A, Iacobuzio-Donahue CA, et al. An analysis of genetic heterogeneity in untreated cancers. Nat Rev Cancer. 2019;19(11):639-50.
8. Krishna A, Meynert A, Dolt KS, Kelder M, Mesropian A, Ewing A, et al. Mutational scanning reveals oncogenic CTNNB1 mutations have diverse effects on signaling. Nat Genet. 2026.
9. Kinnersley B, Sud A, Everall A, Cornish AJ, Chubb D, Culliford R, et al. Analysis of 10,478 cancer genomes identifies candidate driver genes and opportunities for precision oncology. Nat Genet. 2024;56(9):1868-77.
10. Kurnit KC, Kim GN, Fellman BM, Urbauer DL, Mills GB, Zhang W, et al. CTNNB1 (beta-catenin) mutation identifies low grade, early stage endometrial cancer patients at increased risk of recurrence. Mod Pathol. 2017;30(7):1032-41.
11. Yaeger R, Chatila WK, Lipsyc MD, Hechtman JF, Cercek A, Sanchez-Vega F, et al. Clinical Sequencing Defines the Genomic Landscape of Metastatic Colorectal Cancer. Cancer Cell. 2018;33(1):125-36 e3.
12. Ahn SM, Jang SJ, Shim JH, Kim D, Hong SM, Sung CO, et al. Genomic portrait of resectable hepatocellular carcinomas: implications of RB1 and FGF19 aberrations for patient stratification. Hepatology. 2014;60(6):1972-82.
13. Bashashati A, Ha G, Tone A, Ding J, Prentice LM, Roth A, et al. Distinct evolutionary trajectories of primary high-grade serous ovarian cancers revealed through spatial mutational profiling. J Pathol. 2013;231(1):21-34.
14. Al Bakir M, Reading JL, Gamble S, Rosenthal R, Uddin I, Rowan A, et al. Clonal driver neoantigen loss under EGFR TKI and immune selection pressures. Nature. 2025;639(8056):1052-9.
