# Supplementary material for: Integrated Single-Cell Whole-Genome Sequencing and Spatial Transcriptomics Reveal Intratumoral Heterogeneity in Ovarian Cancer
Source: Cancer Res Commun. 2026 May 4;6(5):1020–35. doi: 10.1158/2767-9764.CRC-25-0795 (PMC13137417; doi:10.1158/2767-9764.CRC-25-0795)
Supplement: Supplementary Figure 1 — Clustering of scWGS copy number data [file crc-25-0795_supplementary_figure_1_suppsf1.pdf]

Supplementary Figure 1 – Clustering of scWGS copy number data

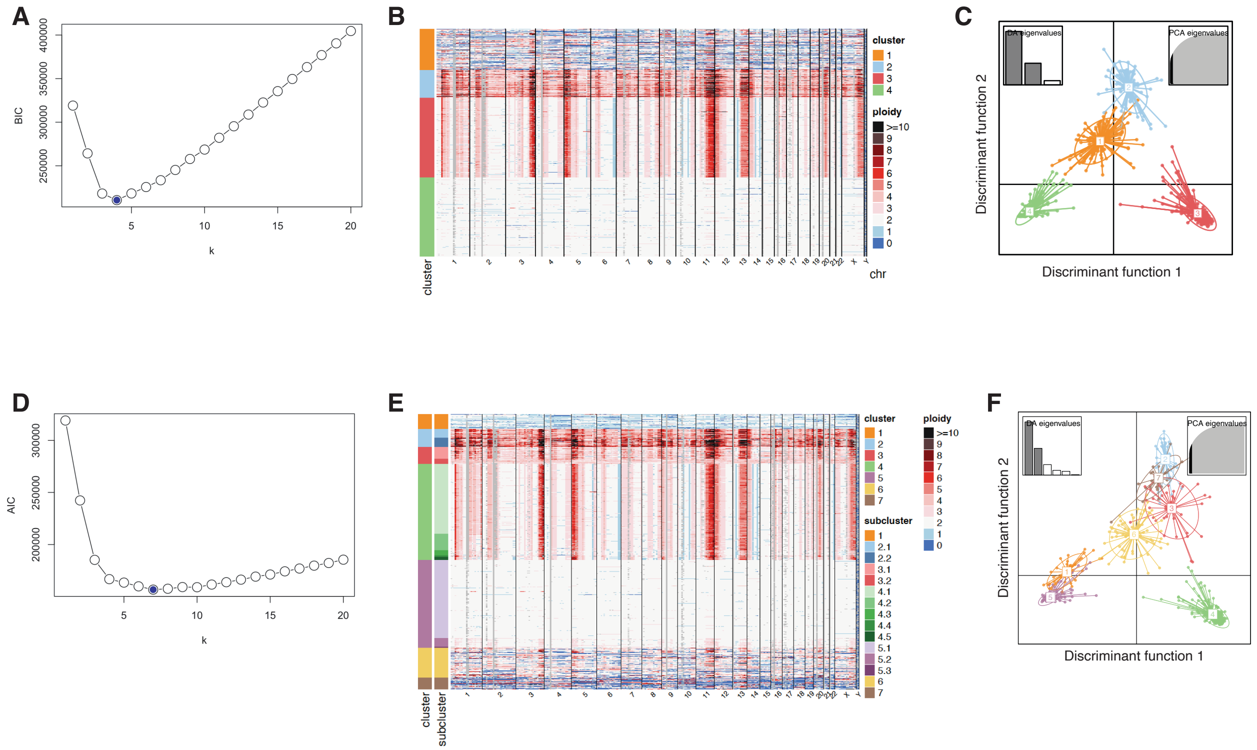

(A) Bayesian Information Criterion (BIC) of clustering solutions up to k = 20 for sample OV440. (B) Single cell copy number heatmap reflecting clustering of OV440 based on k = 4, as informed by BIC. (C) Discriminant analysis of principal components applied to k = 4 clusters of OV440. (D) Akaike Information Criterion (AIC) of clustering solutions up to k = 20 for sample OV440. (E) Single cell copy number heatmap reflecting clustering of OV440 based on k = 7, as informed by AIC. Clusters 1, 6, and 7 were excluded from further analysis due to degradation of DNA. (F) Discriminant analysis of principal components applied to k = 7 clusters of OV440.
