# Supplementary material for: Integrated Single-Cell Whole-Genome Sequencing and Spatial Transcriptomics Reveal Intratumoral Heterogeneity in Ovarian Cancer
Source: Cancer Res Commun. 2026 May 4;6(5):1020–35. doi: 10.1158/2767-9764.CRC-25-0795 (PMC13137417; doi:10.1158/2767-9764.CRC-25-0795)
Supplement: Supplementary Figure 2 — Copy number and LOH at chromosomes 13 and 17 in OV440 [file crc-25-0795_supplementary_figure_2_suppsf2.pdf]

Supplementary Figure 2 – Copy number and LOH at chromosomes 13 and 17 in OV440

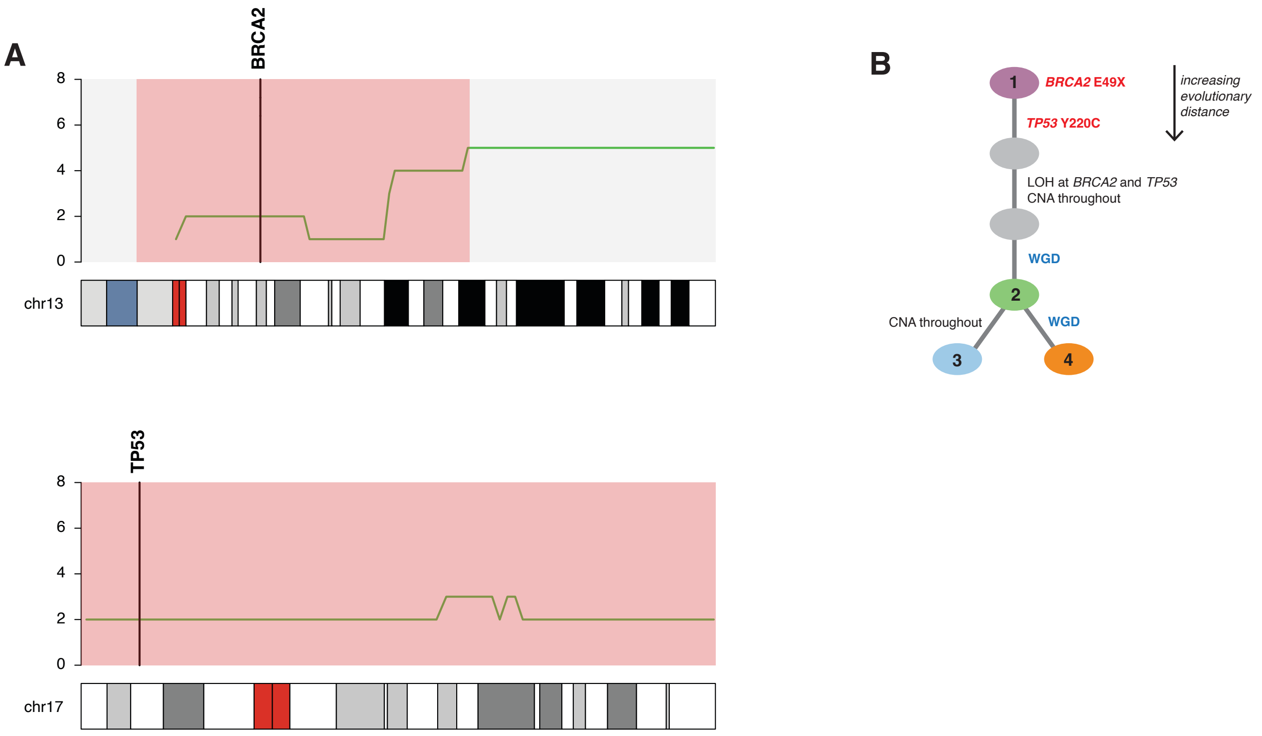

(A) Consensus copy number for cluster 2 of OV440 at 1 Mb resolution at chromosomes 13 and 17. Red shading indicates regions of loss of heterozygosity (LOH). Locations of *BRCA2* and *TP53* are annotated. (B) Hypothesized evolutionary trajectory of OV440, which includes a somatic TP53 mutation as the earliest event, followed by widespread LOH and whole genome doubling (WGD).
