# Supplementary material for: Integrated Single-Cell Whole-Genome Sequencing and Spatial Transcriptomics Reveal Intratumoral Heterogeneity in Ovarian Cancer
Source: Cancer Res Commun. 2026 May 4;6(5):1020–35. doi: 10.1158/2767-9764.CRC-25-0795 (PMC13137417; doi:10.1158/2767-9764.CRC-25-0795)
Supplement: Supplementary Figure 3 — Copy number inferred from OV440 ST data [file crc-25-0795_supplementary_figure_3_suppsf3.pdf]

Supplementary Figure 3 – Copy number inferred from OV440 ST data

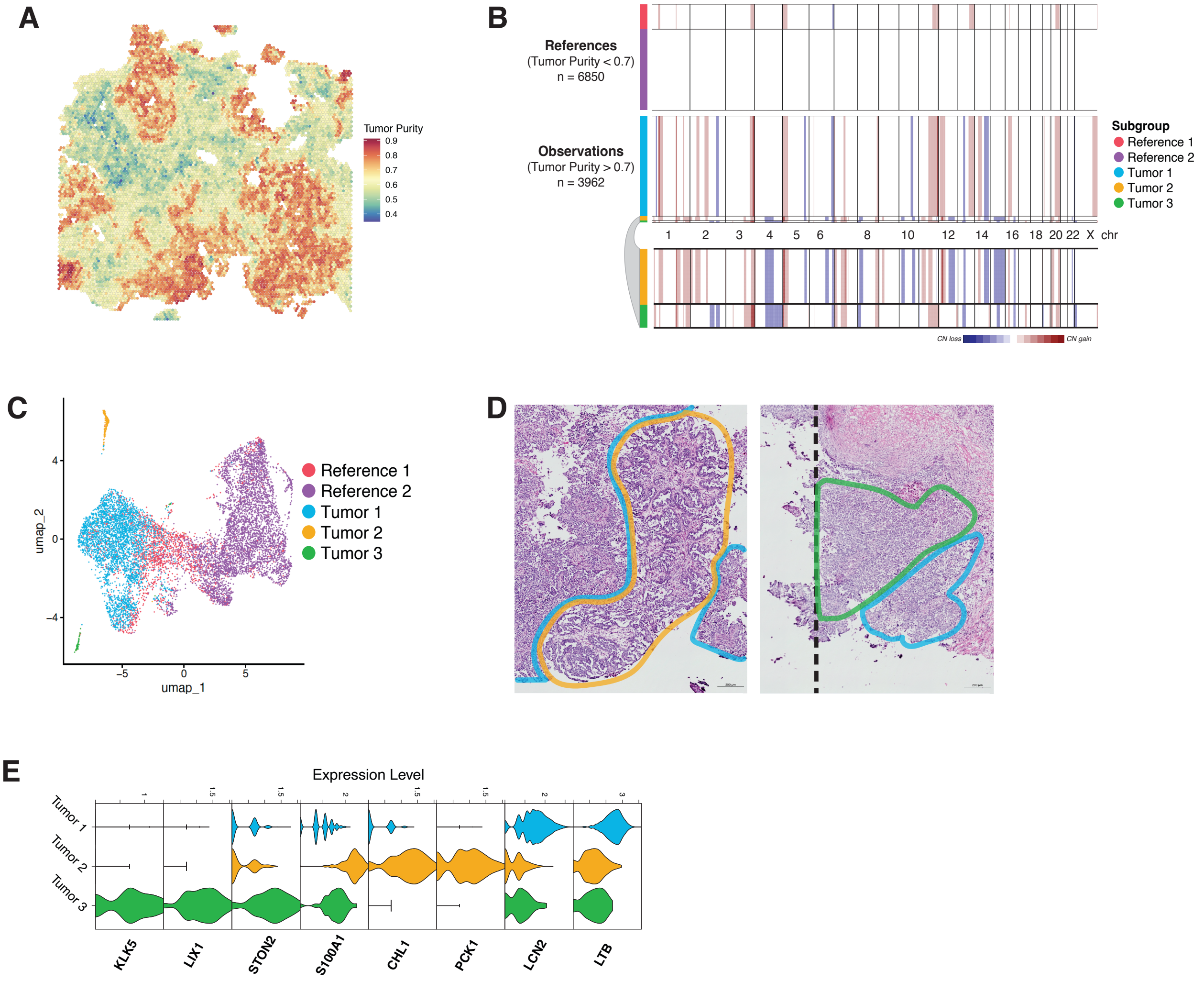

(A) Tumor purity as determined by ESTIMATE, which was used to determine reference and observation annotations for inferCNV. (B) Full inferCNV results for OV440. Subclusters were determined by Leiden clustering and are annotated on the left bar. HMM-based copy number prediction was performed at the subcluster level. Tumors 2 and 3 are expanded beneath the plot for clarity. (C) Subgroups identified in (C) mapped in low-dimensional space. Reference 2 was determined to likely comprise tumor-diploid mixtures at the tumor-stroma interface. (D) Magnified regions corresponding to the tumor subgroups identified. Tumor 2 displays a papillary growth pattern, tumor 3 displays a micropapillary growth pattern, and tumor 1 displays both micropapillary and solid growth patterns. Scale bar represents 200 microns. (E) Log-transformed gene expression of select genes differentially expressed between clusters.
