# Supplementary material for: Integrated Single-Cell Whole-Genome Sequencing and Spatial Transcriptomics Reveal Intratumoral Heterogeneity in Ovarian Cancer
Source: Cancer Res Commun. 2026 May 4;6(5):1020–35. doi: 10.1158/2767-9764.CRC-25-0795 (PMC13137417; doi:10.1158/2767-9764.CRC-25-0795)
Supplement: Supplementary Figure 4 — TP53 mutations in HGSOC samples [file crc-25-0795_supplementary_figure_4_suppsf4.pdf]

Supplementary Figure 4 – TP53 mutations in HGSOC samples

A

OV440 cluster 1

OV440 cluster 2

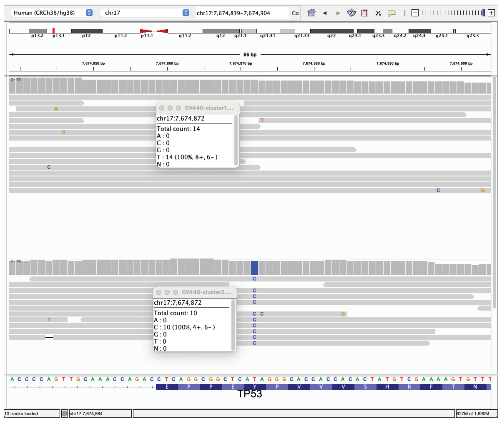

TP53 Y220C

B

OV594 cluster 1

OV594 cluster 2

OV594 cluster 3

OV594 cluster 4

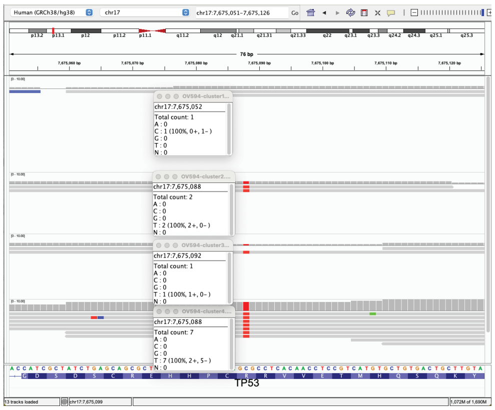

TP53 R175H

C

OV773 cluster 1

OV773 cluster 2

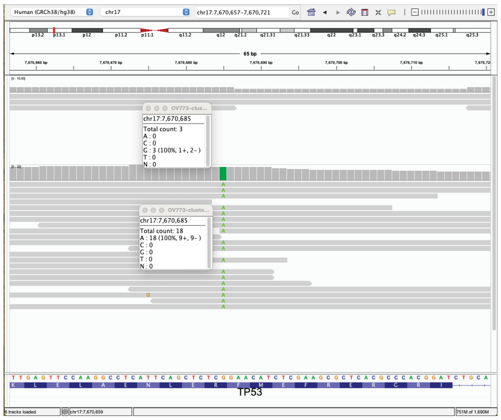

TP53 R342X

The clonal *TP53* mutations in samples (A) OV440, (B) OV594, and (C) OV773.
