# Supplementary material for: Integrated Single-Cell Whole-Genome Sequencing and Spatial Transcriptomics Reveal Intratumoral Heterogeneity in Ovarian Cancer
Source: Cancer Res Commun. 2026 May 4;6(5):1020–35. doi: 10.1158/2767-9764.CRC-25-0795 (PMC13137417; doi:10.1158/2767-9764.CRC-25-0795)
Supplement: Supplementary Figure 5 — OV150 somatic KRAS mutation [file crc-25-0795_supplementary_figure_5_suppsf5.pdf]

Supplementary Figure 5 – OV150 somatic KRAS mutation

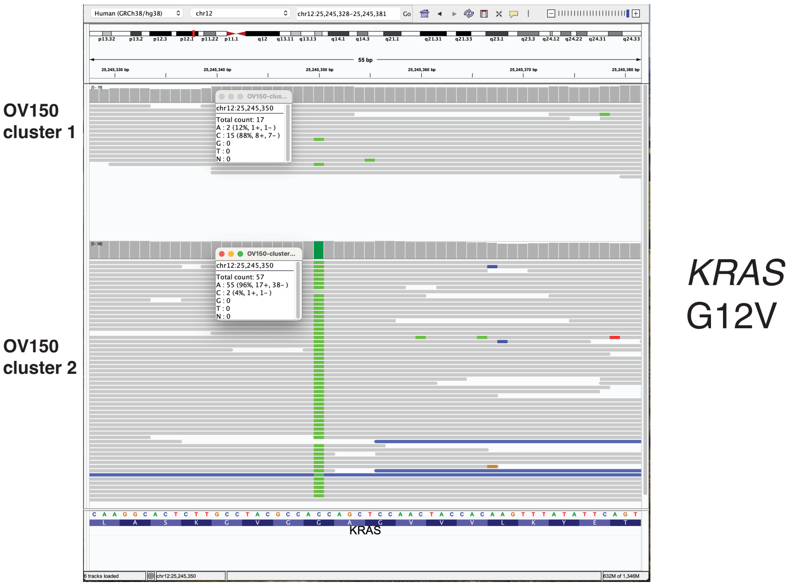

The clonal *KRAS* mutations in OV150 visualized with IGV. The two reads in cluster 1 with the mutation belong to cell 570, which is a pseudodiploid cell described in Figure 4.
