# Supplementary material for: Integrated Single-Cell Whole-Genome Sequencing and Spatial Transcriptomics Reveal Intratumoral Heterogeneity in Ovarian Cancer
Source: Cancer Res Commun. 2026 May 4;6(5):1020–35. doi: 10.1158/2767-9764.CRC-25-0795 (PMC13137417; doi:10.1158/2767-9764.CRC-25-0795)
Supplement: Supplementary Figure 6 — Loadings corresponding to DAPC analysis in Figure 3 [file crc-25-0795_supplementary_figure_6_suppsf6.pdf]

Supplementary Figure 6 – Loadings corresponding to DAPC analysis in Figure 3

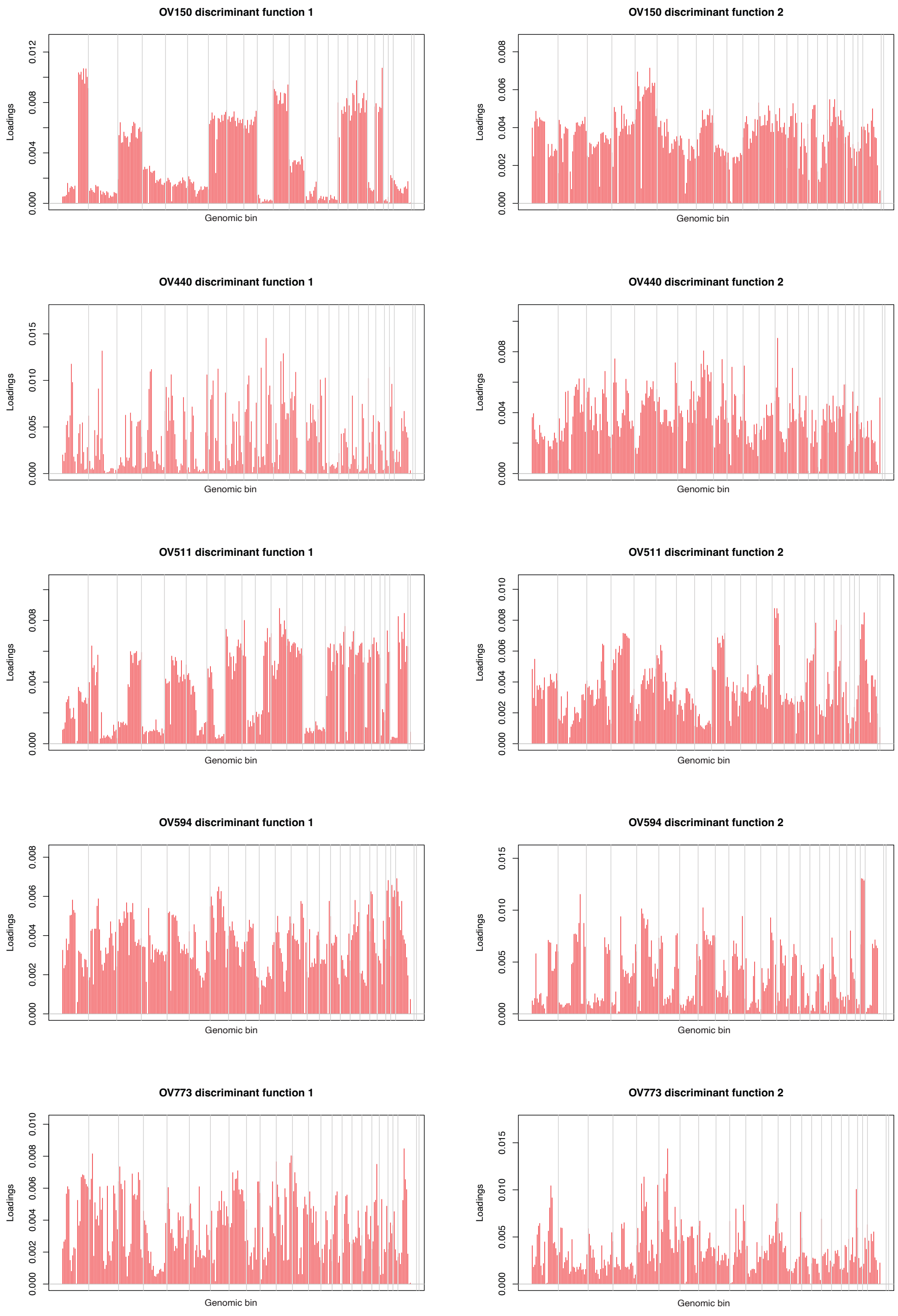

Loadings corresponding to discriminant functions 1 and 2, as presented in main Figure 3, for each sample. The x-axis represents 10 Mb genomics bins, and chromosome boundaries are delineated by vertical lines.
