# Supplementary material for: Integrated Single-Cell Whole-Genome Sequencing and Spatial Transcriptomics Reveal Intratumoral Heterogeneity in Ovarian Cancer
Source: Cancer Res Commun. 2026 May 4;6(5):1020–35. doi: 10.1158/2767-9764.CRC-25-0795 (PMC13137417; doi:10.1158/2767-9764.CRC-25-0795)
Supplement: Supplementary Figure 7 — Chromosome 17 LOH in OV440 [file crc-25-0795_supplementary_figure_7_suppsf7.pdf]

Supplementary Figure 7 – Chromosome 17 LOH in OV440

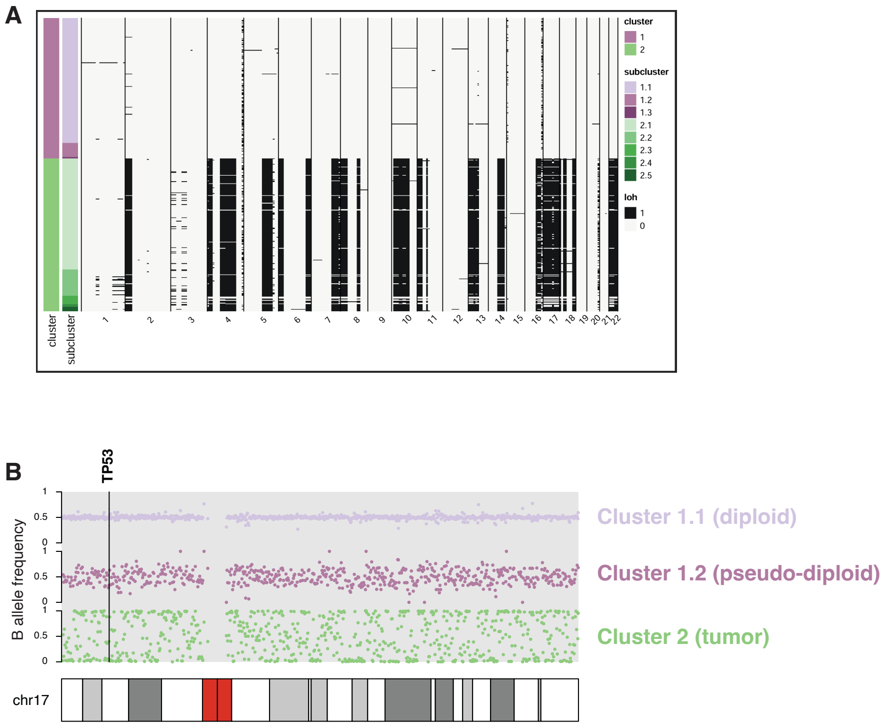

(A) Loss of heterozygosity in sample OV440 inferred from single-cell allele specific copy number determined by CHISEL. Black shading represents LOH. (B) B allele frequencies across chromosome 17 for clusters of OV440.
