# Supplementary material for: Integrated Single-Cell Whole-Genome Sequencing and Spatial Transcriptomics Reveal Intratumoral Heterogeneity in Ovarian Cancer
Source: Cancer Res Commun. 2026 May 4;6(5):1020–35. doi: 10.1158/2767-9764.CRC-25-0795 (PMC13137417; doi:10.1158/2767-9764.CRC-25-0795)
Supplement: Supplementary Figure 8 — Evolution of OV150 subclusters 2.4 and 2.5 [file crc-25-0795_supplementary_figure_8_suppsf8.pdf]

Supplementary Figure 8 – Evolution of OV150 subclusters 2.4 and 2.5

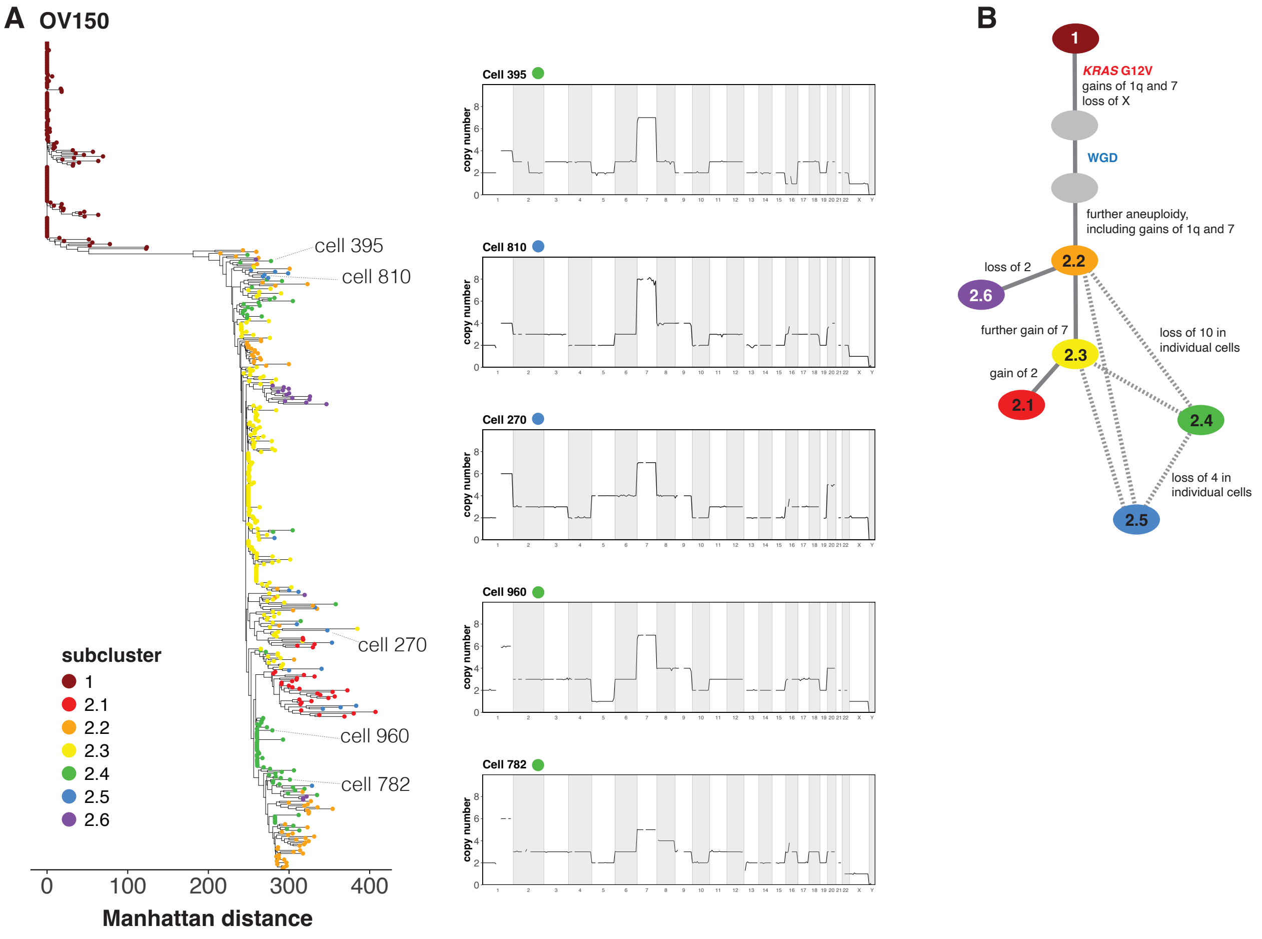

(A) Single cell phylogeny of OV150 (also presented in Figure 4G) annotated with representative cells from subclusters 2.4 and 2.5 and their copy number plots. (B) Hypothesized evolutionary trajectory for sample OV150. KRAS mutation is the clonal driver, and is accompanied by early CNAs in chrs 1q, 7, and X. Dotted lines indicate that clusters 2.4 and 2.5 may be derived from multiple preceding clusters, as a result of aneuploidy in individual cells.
