# Supplementary material for: Integrated Single-Cell Whole-Genome Sequencing and Spatial Transcriptomics Reveal Intratumoral Heterogeneity in Ovarian Cancer
Source: Cancer Res Commun. 2026 May 4;6(5):1020–35. doi: 10.1158/2767-9764.CRC-25-0795 (PMC13137417; doi:10.1158/2767-9764.CRC-25-0795)
Supplement: Supplementary Figure 9 — Spatial NRF1 and NFE2L2 expression in OV594 [file crc-25-0795_supplementary_figure_9_suppsf9.pdf]

Supplementary Figure 9 – Spatial NRF1 and NFE2L2 expression in OV594

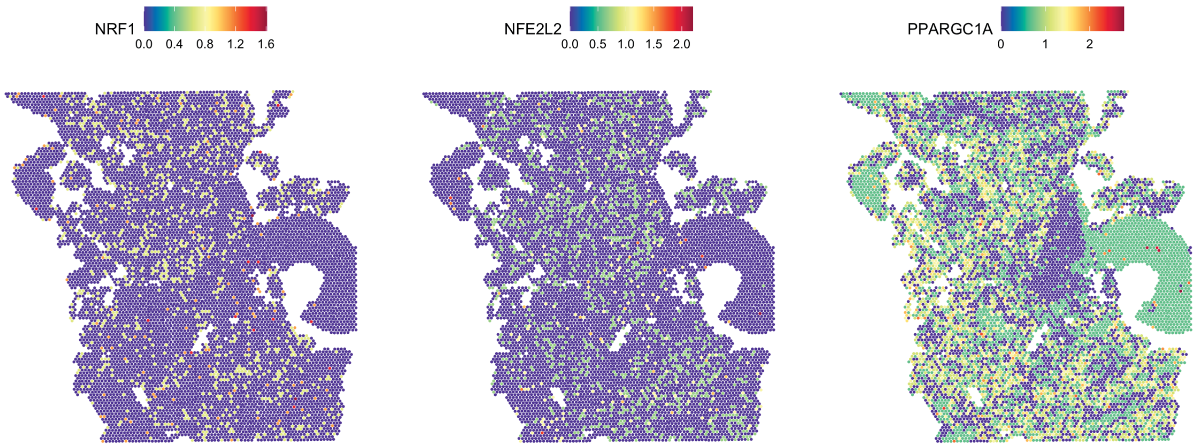

Spatially mapped log-transformed gene expression of *NRF1*, *NFE2L2*, and *PPARGC1A* in sample OV594.
