# Supplementary material for: Integrated Single-Cell Whole-Genome Sequencing and Spatial Transcriptomics Reveal Intratumoral Heterogeneity in Ovarian Cancer
Source: Cancer Res Commun. 2026 May 4;6(5):1020–35. doi: 10.1158/2767-9764.CRC-25-0795 (PMC13137417; doi:10.1158/2767-9764.CRC-25-0795)
Supplement: Supplementary Figure 10 — Shared copy number events in OV511 clones [file crc-25-0795_supplementary_figure_10_suppsf10.pdf]

Supplementary Figure 10 – Shared copy number events in OV511 clones

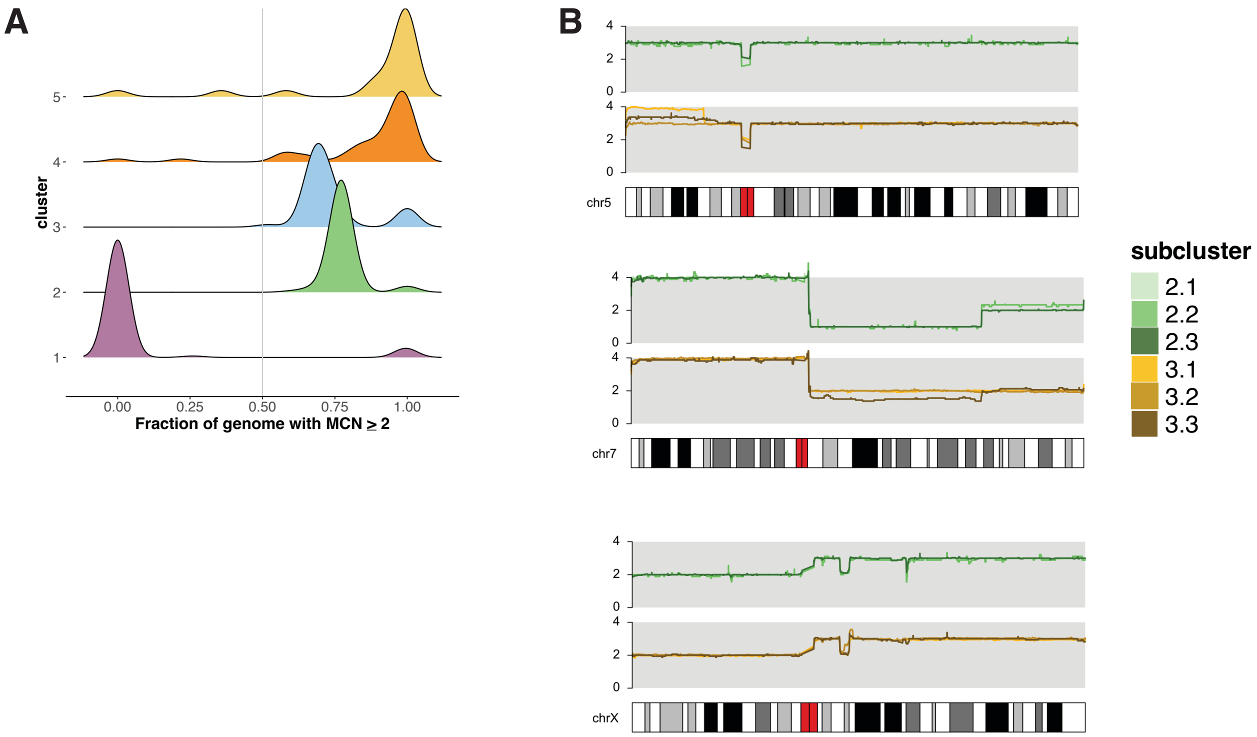

(A) Evidence for whole genome doubling in sample OV511, defined as greater than 50% of the genome with a major allele copy number (MCN) of 2 or greater . (B) Average copy number line plots for sample OV511 illustrating shared CNA at three representative chromosomes. Copy number is displayed on the y-axis and plotted at 20Kb resolution.
