# Supplementary material for: Integrated Single-Cell Whole-Genome Sequencing and Spatial Transcriptomics Reveal Intratumoral Heterogeneity in Ovarian Cancer
Source: Cancer Res Commun. 2026 May 4;6(5):1020–35. doi: 10.1158/2767-9764.CRC-25-0795 (PMC13137417; doi:10.1158/2767-9764.CRC-25-0795)
Supplement: Supplementary Figure 11 — OV511 somatic variants [file crc-25-0795_supplementary_figure_11_suppsf11.pdf]

Supplementary Figure 11 – OV511 somatic variants

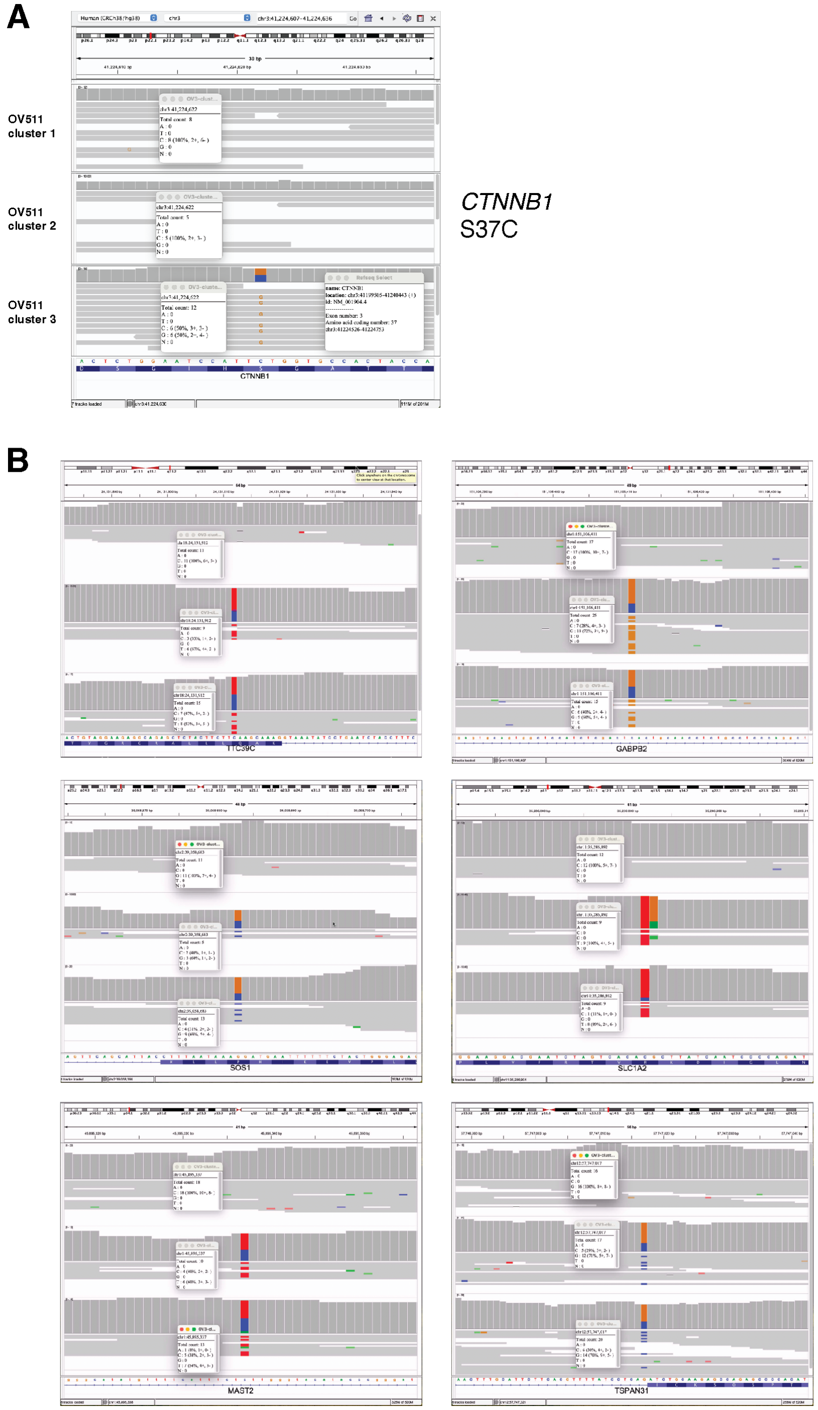

(A) The putative *CTNNB1* truncal driver mutation in sample OV511 visualized in IGV. (B) Select somatic passenger mutations from Figure 7D shared by clusters 2 and 3 in sample OV511.
