# Supplementary material for: Integrated Single-Cell Whole-Genome Sequencing and Spatial Transcriptomics Reveal Intratumoral Heterogeneity in Ovarian Cancer
Source: Cancer Res Commun. 2026 May 4;6(5):1020–35. doi: 10.1158/2767-9764.CRC-25-0795 (PMC13137417; doi:10.1158/2767-9764.CRC-25-0795)
Supplement: Supplementary Figure 12 — LOH at CTNNB1 SNPs [file crc-25-0795_supplementary_figure_12_suppsf12.pdf]

Supplementary Figure 12 – LOH at CTNNB1 SNPs

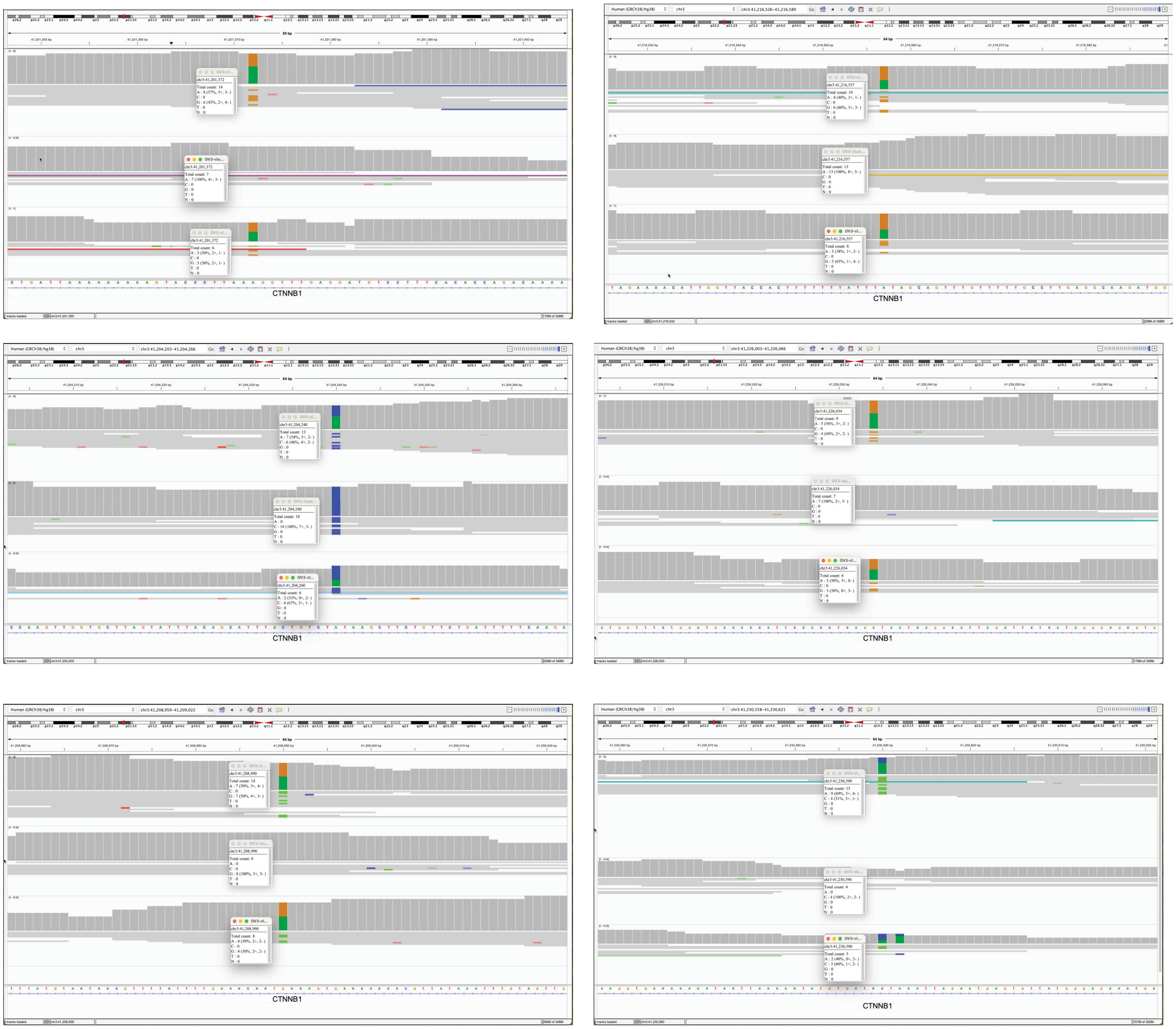

Heterozygous germline SNPs within *CTNNB1* support LOH in cluster 2 of OV511. Tracks are in order from top to bottom: cluster 1, cluster 2, cluster 3
