# Supplementary material for: Integrated Single-Cell Whole-Genome Sequencing and Spatial Transcriptomics Reveal Intratumoral Heterogeneity in Ovarian Cancer
Source: Cancer Res Commun. 2026 May 4;6(5):1020–35. doi: 10.1158/2767-9764.CRC-25-0795 (PMC13137417; doi:10.1158/2767-9764.CRC-25-0795)
Supplement: Supplementary Figure 13 — Positional gene set enrichment in OV511 [file crc-25-0795_supplementary_figure_13_suppsf13.pdf]

Supplementary Figure 13 – Positional gene set enrichment in OV511

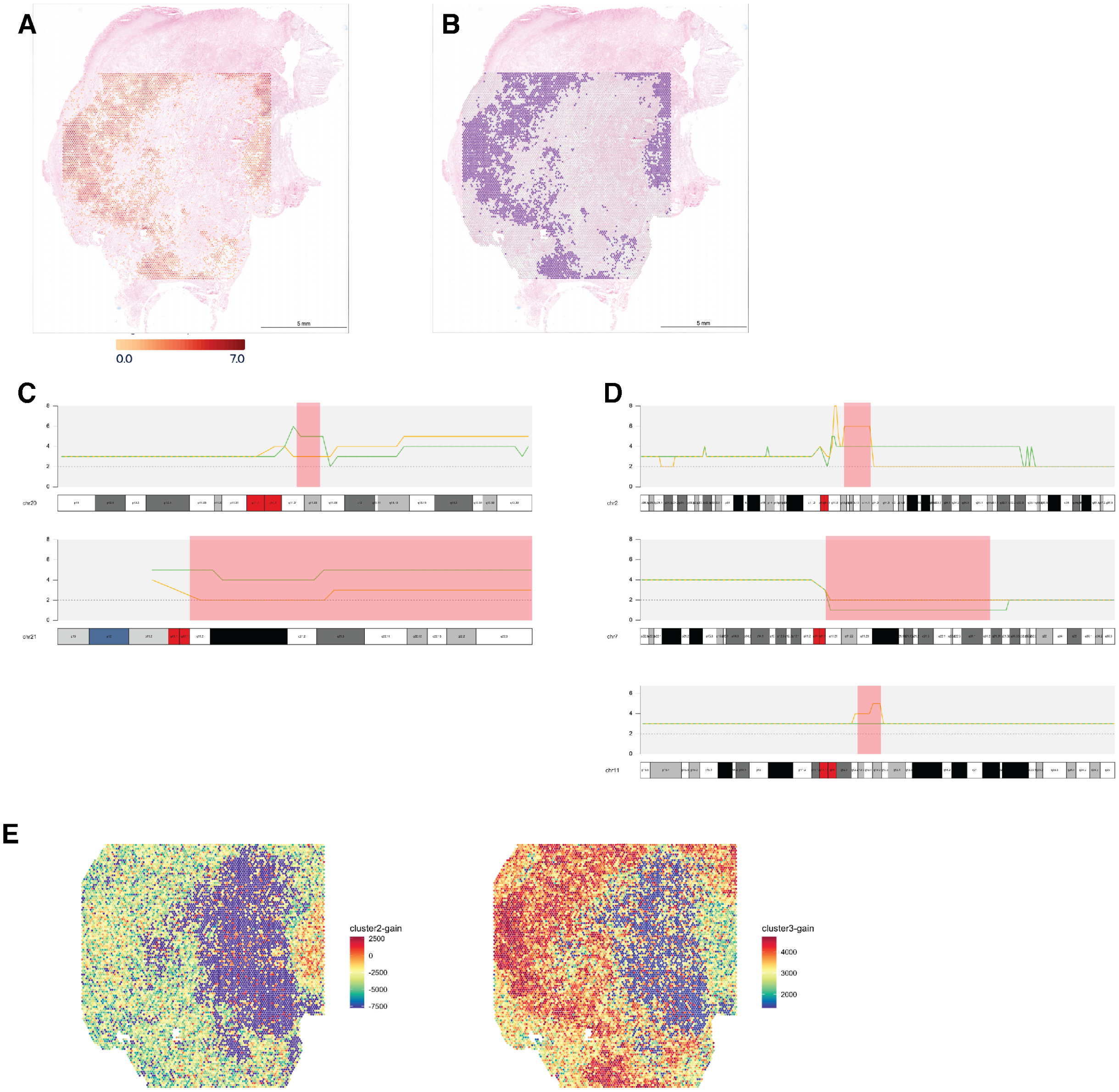

(A) Aggregate expression of *EPCAM*, *KRT7*, and *KRT8* in OV511. Scale bar represents log2 of the summed expression. (B) Spots passing the filtering threshold of log2 summed expression > 1.5. We consider these spots to be comprised mostly of epithelial cells. (C) Regions of the genome utilized in the positional gene set for cluster 2. All unique canonical genes in these regions were exported from UCSC genome browser. (D) Regions of the genome utilized in the positional gene set for cluster 3. (E) Spatially mapped ssGSEA enrichment scores corresponding to the custom positional gene sets for each cluster. Spots passing epithelial filtering (B) were assigned to cluster 2 if the cluster 2-gain score was greater than 2500, and to cluster 3 if the cluster3-gain score was greater than 3000.
