# Supplementary material for: Integrated Single-Cell Whole-Genome Sequencing and Spatial Transcriptomics Reveal Intratumoral Heterogeneity in Ovarian Cancer
Source: Cancer Res Commun. 2026 May 4;6(5):1020–35. doi: 10.1158/2767-9764.CRC-25-0795 (PMC13137417; doi:10.1158/2767-9764.CRC-25-0795)
Supplement: Supplementary Figure 14 — Histology of OV511 clones [file crc-25-0795_supplementary_figure_14_suppsf14.pdf]

Supplementary Figure 14 – Histology of OV511 clones

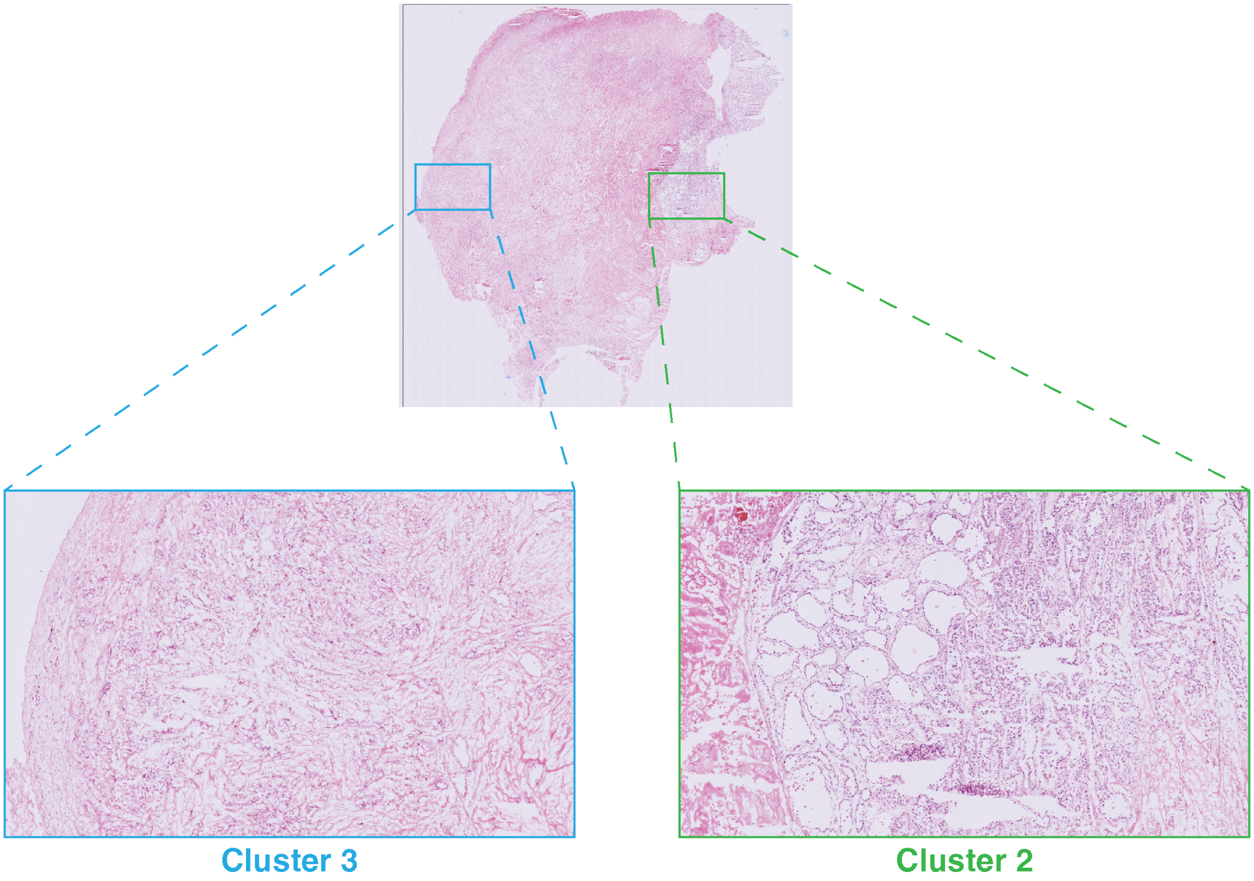

Magnified H&E-stained tissue from sample OV511. The indicated regions were determined to correspond to the labeled clusters by positional gene set enrichment.
